# Model Predictive Controller Based on Online Obtaining of Softness Factor and Fusion Velocity for Automatic Train Operation

**DOI:** 10.3390/s20061719

**Published:** 2020-03-19

**Authors:** Longda Wang, Xingcheng Wang, Zhao Sheng, Senkui Lu

**Affiliations:** 1 School of Marine Electrical Engineering, Dalian Maritime University, Dalian 116026, China; wanglongda@dlmu.edu.cn (L.W.); dlm@dlmu.edu.cn (S.L.); 2 School of Electronic and Information Engineering, Beijing Jiaotong University, Beijing 100044, China; zhaosheng@bjtu.edu.cn

**Keywords:** model predictive controller, automatic train operation, softness factor, fusion velocity, online obtaining, hardware-in-the-loop simulation

## Abstract

This paper develops an improved model predictive controller based on the online obtaining of softness factor and fusion velocity for automatic train operation to enhance the tracking control performance. Specifically, the softness factor of the improved model predictive control algorithm is not a constant, conversely, an improved online adaptive adjusting method for softness factor based on fuzzy satisfaction of system output value and velocity distance trajectory characteristic is adopted, and an improved whale optimization algorithm has been proposed to solve the adjustable parameters; meanwhile, the system output value for automatic train operation is not sampled by a normal speed sensor, on the contrary, an improved online velocity sampled method for the system output value based on a fusion velocity model and an intelligent digital torque sensor is applied. In addition, the two improved strategies proposed take the real-time storage and calculation capacities of the core chip of the controller into account. Therefore, the proposed improved strategies (I) have good performance in tracking precision, (II) are simple and easily conducted, and (III) can ensure the accomplishing of computational tasks in real-time. Finally, to verify the effectiveness of the improved model predictive controller, the Matlab/simulink simulation and hardware-in-the-loop simulation (HILS) are adopted for automatic train operation tracking control, and the tracking control simulation results indicate that the improved model predictive controller has better tracking control effectiveness compared with the existing traditional improved model predictive controller.

## 1. Introduction

The urban rail transit system with automatic train operation system has the advantages of safety, stability, economy, and comfort, and it has become one of the most popular and efficient means of the urban public transportation [1]. The tracking control functional module makes the velocity trajectory track at the optimal target speed obtained by the upper-layer optimal loop, and according to the appropriate and efficient tracking control algorithm, it is an indispensable crucial system and necessary to ensure optimal safety, comfort, energy-efficiency, punctuality, and parking accuracy for train operation process, which requires the corresponding algorithm to possess good control performance [2]. Therefore, aiming at improving the multi-objective performance index of the train operation process, an automatic train operation system has been developed rapidly and is widely applied in urban rail trains operation [3,4,5]. Meanwhile, various improved intelligent optimization control algorithms have been proposed and applied for the automatic train operation system [6,7,8].

In recent years, many improved algorithms have been applied in the automatic train operation tracking control field, such as robust adaptive automatic control, model predictive control, online learning, iterative learning control, matter-element model, etc. [9,10,11,12,13]. An online approximation-based robust adaptive automatic train control method is proposed for the automatic train operation (ATO) system [9]. A fuzzy model predictive control approach is proposed to provide locomotive operation instructions for mainline railways continuously, and extensive simulations show that the proposed approach can provide sufficient solution optimality in reasonable computational time and energy consumption in train operations is reduced [10]. A novel online learning control strategy is proposed to solve the train automatic stop control (TASC) problem [11]. An iterative learning control based on automatic train operation is proposed to deal with the trajectory tracking control problem under certain velocity constrains [12]. Matter-element theory is applied to the established models to optimize speed trajectory for achieving multi-objective optimization, and the relative performance indices weighting is determined in different stages so that the more satisfied decision speeds could be calculated with the goodness evaluation method [13]. The above research can improve the tracking control performance of the traditional control algorithms.

Among numerous algorithms, Model Predictive Control (MPC) is one of the most effective control algorithms, which is characterized by good robustness, fast tracking speed, accurate tracking target speed, etc. [14]. A linear time-varying MPC is used to obtain the power split between the combustion engine and electrical machines and the system operating points at each sample time [15]. A coordinated energy dispatch based on Distributed model predictive control (DMPC) is proposed, and the corresponding simulation results show the effectiveness of the proposed method [16]. A co-design of the self-triggered mechanism and distributed model predictive control (DMPC) is proposed to achieve the cooperative objectives while efficiently exploiting communication network [17]. A model predictive control-based droop current regulator to interface PV in smart dc distribution systems is proposed [18]. From the various model predictive control algorithms, the dynamic matrix control model predictive control (DMC MPC) is an effective algorithm among them due to its characteristics of strong robustness, fast tracking speed, high precision for tracking control, avoiding the parameter identification for the transfer function model, and solving the problem of delay process effectively. A new method that linearizes the RC equivalent circuit model and predicts available battery power according to original Dynamic Matrix Control algorithm is proposed [19]. An application of dynamic matrix control (DMC) to a drum-type boiler–turbine system is proposed [20]. Of particular note is the use of the improved DMC MPC for automatic train operation tracking control scenario [21].

It is necessary to conduct further study on the basis of previous research findings, and key parameters adjusting and improving the sampling accuracy should be taken seriously. A method for calculating the traction characteristics of a traction motor is proposed [22]. A new method to identify the train key design variables against the running performance indicators based on the sensitivity analysis is proposed, which in turn bases itself on simulation-oriented surrogate models [23]. A novel adaptive sampling algorithm for power management in the automated monitoring of the quality of water in an environment is devised and applied [24].

Traditional simulations based on a pure software environment cannot truly reflect the actual automatic train operation process, and the situation representing the actual automatic train operation experiment is difficult to implement because it is expensive, has restricted experimental conditions, high construction difficulties, and high security protection requirements. Hardware-in-the-loop simulation (HILS) is a new simulation technology for solving this difficult issue [25,26]. At present, numerous relative research findings have achieved improvements in the traction control system [27,28].

An improved model predictive controller based on online obtaining of softness factor and fusion velocity for automatic train operation is developed. The following summarizes the main contributions of this paper.

(I) An improved whale optimization algorithm (IWOA) based on the Tchebycheff decomposition method, convergence factor nonlinear decline, and genetic evolution measurement is proposed to solve the optimization of the softness factor adaptive adjusting parameters appropriately.
(II) Aiming at improving the tracking control performance for automatic train operation, an improved model predictive controller based on online obtaining of the softness factor and fusion velocity is developed for automatic train operation tracking control effectively.
(III) To further verify the effectiveness of the developed model predictive control controller, the scenario about rail transit line No.12 in Dalian, China is chosen for simulation test. The results of the Matlab simulation and hardware-in-the-loop simulation (HILS) show that the tracking controller proposed in this paper has good tracking control performance.

The paper is organized as follows. Section 2 introduces the model predictive controller for automatic train operation tracking control. Section 3 illustrates the improved DMC model predictive controller based on online obtaining of softness factor and fusion velocity developed in this paper. Section 4 provides the Matlab/simulation results and hardware-in-the-loop simulation (HILS) results to illustrate the proposed method. Section 5 concludes this article.

## 2. Model Predictive Controller for Automatic Train Operation Tracking Control

### 2.1. Evaluation Index for Automatic Train Operation Tracking Control

The integral of time multiplied by the absolute value of error (ITAE) is the frequently used evaluation index for tracking control performance [29]. The specific formula for the evaluation index ITAE is as follows,

(1) $$\begin{array}{c} ITAE=\int t\left|e\left(t\right)\right|dt \end{array}$$

where *t* represents the sample time of control process, and $\left|e\left(t\right)\right|$ represents the absolute value of error between target speed and actual tracking control speed.

As automatic train operation has its own unique characteristics and requirements, the multi-objective performance index is more appropriative, and it used universally. The computation model of multi-objective performance index $P_{k}$ is as follows,

(2) Pk=∑i=14ωi×fi−min(fi)max(fi)−min(fi)×fi(f1,f2,f3,f4)=(Δs,Δt,KJerk,E)Mvdvds=Fu,v−Rv,s−Bu,vdtds=1vvs≤vlimsRv,s=r(v)+RlsΔs=sz−D<ΔsmaxΔt=T¯−Tr<ΔtmaxKJerk=∫Δads∫ΔadsDDE=∫Ma−Rds

where $\omega_{i}$ represents the index importance weight factor ($\overset{k}{\underset{i=1}{\sum }}{\omega^{\prime }}_{i}=1$), which reflects the relative importance of the *i* th optimization index; *t* represents the actual running time of the train; *s* represents the actual position of the train; *a* represents the actual acceleration of the train; $\left|\Delta a\right|$ represents the actual impingement rate of the train; *M* is the mass of the train; $F_{t}\left(u,v\right)$ and $B_{r}\left(u,v\right)$ are the traction force and braking force of the current velocity, respectively; $R\left(v,s\right)$ is the resistance of the train determined by the current speed and line position; $s_{z}$ is the terminal position; $T_{r}$ is the actual running time; *D* is the actual running distance; $v\left(s\right)$ represents the instantaneous velocity in the position *s*; $\bar{T}$ represents the prospective running time; $v_{lim}\left(s\right)$ represents the upper limit velocity in the position *s*; $\Delta s_{\max}$ represents the allowable maximum parking error; $\Delta t_{\max}$ represents the allowable maximum time error; Δs and Δt represent the actual parking error and time error, respectively; *u* represents the train control quantity; $K_{Jerk}$ represents the comfort performance index; and *E* represents the energy consumption during the train operation process [2,30].

In addition, security index should be taken into account as well. Traveling over the velocity limit is the main risk and non-negligible factors can cause an unsafe environment. The computation formula of security index $K_{safe}$ is as follows,

(3) minKsafeKsafe=∑is=1snYSissnYS(is)={0vis>vlimis1vis≤vlimis

where is represents the index of sampling point, $YS\left(is\right)$ represents the security evaluation value of the is-th sampling point, and sn represents the number of sampling points [1].

### 2.2. Conventional Dynamic Matrix Control Model Predictive Control

DMC MPC uses three methods, including the DMC predictive model, rolling optimization, and feedback correction, to control the controlled object [31].

#### 2.2.1. DMC Predictive Model

The DMC predictive model is one of significant models for DMC MPC. The unit step response model reflecting the dynamic performance is adopted as the DMC predictive model for controlled object, and the predictive value of system output is obtained by the step response characteristic for controlled object.

If the model length is *N*, then the *N* sampled values of the controlled object unit step response can be used to describe the dynamic response characteristics of the system. The specific calculation formula for the predictive value of system output is as follows,

(4) $$\begin{array}{c} Y_{p}\left(k\right)=Y_{0}\left(k\right)+A\Delta U\left(k\right) \end{array}$$

where $Y_{p}\left(k\right)={\left[y_{p}\left(k+1|k\right),y_{p}\left(k+2|k\right),\ldots ,y_{p}\left(k+N|k\right)\right]}^{T}$ represents the predictive value of system output, $Y_{0}\left(k\right)={\left[y_{0}\left(k+1|k\right),y_{0}\left(k+2|k\right),\ldots ,y_{0}\left(k+N|k\right)\right]}^{T}$ represents the predictive value of predictive model, $\Delta U\left(k\right)={\left[\Delta u\left(k\right),\Delta u\left(k+1\right),\ldots ,\Delta u\left(k+N-1\right)\right]}^{T}$ represents the incremental sequence for control, and *A* represents the dynamic matrix. The specific dynamic matrix *A* and the specific calculation formula for the element of $Y_{p}$ are as follows,

(5) $$A=\left[\begin{array}{c} \begin{array}{c} a_{1} \end{array}\begin{array}{c} 0 \end{array}\begin{array}{c} 0 \end{array}\begin{array}{c} \cdots \end{array}\begin{array}{c} 0 \end{array}\\ \begin{array}{c} a_{2} \end{array}\begin{array}{c} a_{1} \end{array}\begin{array}{c} 0 \end{array}\begin{array}{c} \cdots \end{array}\begin{array}{c} 0 \end{array}\\ \begin{array}{c} \vdots \end{array}\begin{array}{c} \vdots \end{array}\begin{array}{c} \ddots \end{array}\begin{array}{c} \ddots \end{array}\\ \begin{array}{c} a_{N} \end{array}\begin{array}{c} a_{N-1} \end{array}\begin{array}{c} \cdots \end{array}\begin{array}{c} a_{1} \end{array} \end{array}\right]$$

(6) $$\begin{array}{c} y_{p}\left(k+i|k\right)=y_{0}\left(k+i|k\right)+\overset{i}{\underset{j=1}{\sum }}a_{i-j+1}\Delta u\left(k+j-1\right) \end{array}$$

where *i* represents the element index of $Y_{p}$, $i\in \left\{1,2,\ldots ,N\right\}$; *k* represents the initial point of DMC predictive model [31,32].

#### 2.2.2. Rolling Optimization

With the aim of avoiding the violent fluctuations in the control process effectively, it is necessary to make the final output value $y_{f}$ reach to the reference target value $y_{r}$ along the predetermined smooth path by the DMC MPC system, so as to enhance the system robustness. Thus, a popular reference path used in DMC MPC is as follows,

(7) $$\begin{array}{c} y_{f}\left(k+i\right)=\alpha^{i}y\left(k\right)+\left(1-\alpha^{i}\right)y_{r} \end{array}$$

where $y_{f}\left(k+i\right)$ represents the final output value expected, $\alpha^{i}$ represents the *i* th softness factor (0 < $\alpha^{i}$ < 1), $y\left(k\right)$ represents the actual output value of the system, and $y_{r}$ represents the reference target value of the system.

The quadratic rolling optimization object of the system is necessary for rolling optimization. If the predictive length is *M* and control length is *L*, in general, L≤M≤N. The specific quadratic rolling optimization object of system is as follows,

(8) $$\begin{array}{c} \begin{array}{c} J={∥Y_{p}\left(k\right)-Y_{f}\left(k\right)∥}_{Q}^{2}+{∥\Delta U_{L}\left(k\right)∥}_{R}^{2}\\ =\overset{M}{\underset{i=1}{\sum }}q_{i}\left[y_{p}\left(k+i|k\right)-y_{f}\left(k+i\right)\right]+\overset{L}{\underset{i=1}{\sum }}r_{i}\Delta u\left(k+i-1\right) \end{array} \end{array}$$

where $Y_{f}\left(k\right)={\left[y_{f}\left(k+1\right),y_{f}\left(k+2\right),\ldots ,y_{f}\left(k+M\right)\right]}^{T}$ represents the control sequence of the system, $R=diag{\left[r_{1},r_{2},\ldots ,r_{L}\right]}^{T}$ represents the weight coefficient matrix of constraint for error revise, $Q\;=\;diag{\left[q_{1},q_{2},\ldots ,q_{M}\right]}^{T}$ represents the weight coefficient matrix of constraint for error increment revise, and diag represents the diagonal matrix.

The necessary condition for obtaining the minimum value of objective function *J* is $\frac{\partial J}{\partial \Delta U_{L}\left(k\right)}=0$ through extreme value theory under unconstrained conditions. Therefore, the control sequence optimal solution can be obtained by rolling optimization. The specific calculation formula of the control sequence optimal solution is as follows.

(9) $$\begin{array}{c} \Delta U_{L}\left(k-1\right)={\left(A^{T}QA+R\right)}^{-1}A^{T}Q\left[Y_{f}\left(k\right)-Y_{0}\left(k\right)\right] \end{array}$$

Then, the actual control quantity u(k) can be obtained. The specific calculation formula of the actual control quantity u(k) is as follows,

(10) $$\begin{array}{c} u\left(k\right)=u\left(k-1\right)+\Delta u\left(k-1\right) \end{array}$$

In the next control period, i.e., the k+1 th control period, the corresponding Δu(k) and u(k+1) can be obtained by the above way. Thus, it can realize the rolling optimization of the actual control quantity in the iterative control process [21,33].

#### 2.2.3. Feedback Correction

Feedback correction is an important component of DMC MPC; it is used to reduce the influence of system disturbance for the control system, so as to achieve the ideal control effectiveness. The specific calculation formula of the error between actual system output value and the predicted output value in the present control period (the *k* th control period) is as follows.

(11) $$\begin{array}{c} e\left(k\right)=y\left(k\right)-y_{p}\left(k\right) \end{array}$$

After feedback correction calculation, the predicted output value can be corrected to certain extent [21,33,34]. The specific corrected calculation formula is as follows,

(12) $$\begin{array}{c} Y_{p2}\left(k\right)=Y_{0}\left(k\right)+A\Delta U\left(k-1\right)+HC \end{array}$$

where *C* represents the error corrected matrix, and its length is *N*; *H* represents the corresponding transformed matrix.

### 2.3. Fuzzy DMC Model Predictive Controller for Automatic Train Operation

Aiming at improving the precision of the automatic train operation tracking control for DMC MPC, using the fuzzy model prediction based on the train operation mechanism is a good choice. The slope and velocity error are the most important train operation information. For example, when the train runs in steep uphill and current velocity is far less than target velocity, the conversion degree for train operation is “PB”, that is, the maximum extent to drew train is adopted to assist the climb by accelerating or keeping the velocity. In this time, if the maximum traction is used according to the intrinsic DMC MPC, the addition traction incremental quantity is not necessary; otherwise, the appropriate addition traction incremental quantity should be used to correct this error. The fuzzy sets are divided into [’N4’,…….,’Z’,…….,’P4’] [10,35]. The specific calculation model for fuzzy model prediction is as follows,

(13) $$\begin{array}{c} \begin{array}{c} u_{f\_p}\left(k\right)=C_{fuzzy1}\left(\omega \left(k\right),e\left(k\right)\right)\\ \Delta u_{f\_p}\left(k\right)=C_{fuzzy2}\left(u_{f\_p}\left(k\right),u\left(k\right)\right)\\ u_{c}\left(k\right)=u\left(k\right)+\Delta u_{f\_p}\left(k\right) \end{array}\; \end{array}$$

where $C_{fuzzy1}$ and $C_{fuzzy2}$ represent the fuzzy inference functions by using two kinds of fuzzy rules, respectively; $u_{f\_p}\left(k\right)$ represents the calculated control quantity by using fuzzy rule about slope and velocity error; $\Delta u_{f\_p}\left(k\right)$ represents the calculated control quantity by using fuzzy rule about control quantity calculated by intrinsic DMC MPC and control quantity calculated by fuzzy logic and train operation information; and $u_{c}\left(k\right)$ represents the final calculated control quantity for the automatic train operation tracking control.

**Figure 1:**
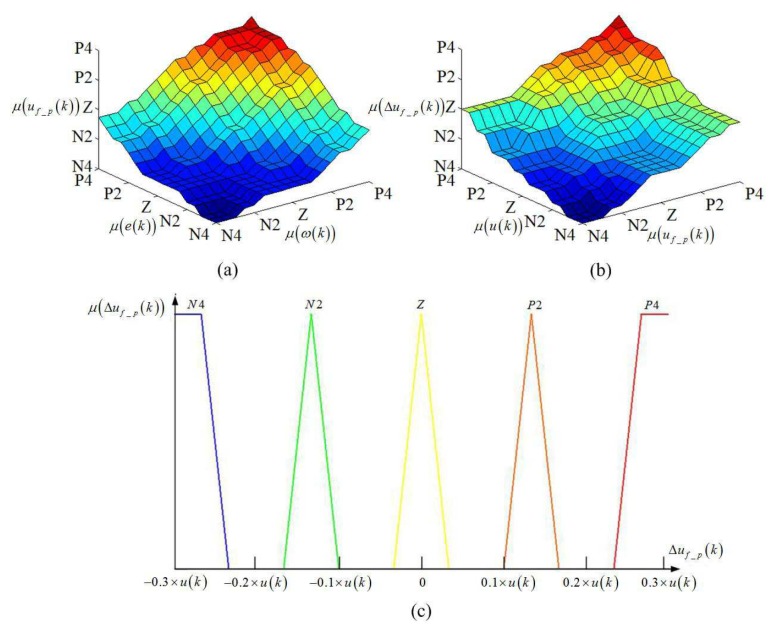
The fuzzy rules for fuzzy model prediction and partial membership function for control quantity. (**a**) Fuzzy rule for train operation information. (**b**) Fuzzy rule for control quantity prediction. (**c**) Partial membership functions for control quantity.

The fuzzy rules for fuzzy model prediction and partial membership function for control quantity are shown in Figure 1.

**Figure 2:**
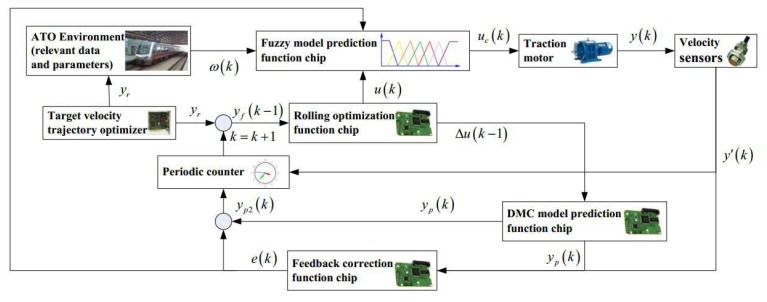
Schematic diagram of the Fuzzy DMC model predictive controller for automatic train operation.

Fuzzy dynamic matrix control model predictive control (Fuzzy DMC MPC) is a control method that considers the step response characteristics and fuzzy logic for the train operation mechanism of the control object. The fuzzy DMC model predictive controller is widely used in automatic train operation due to its characteristics of simple design scheme and high tracking precision. The fuzzy DMC model prediction controller is mainly composed of four function chips (Fuzzy model prediction function chip, DMC model prediction function chip, rolling optimization function chip, and feedback correction function chip), and it is used to realize four function modules of Fuzzy DMC MPC (Fuzzy model prediction, DMC model prediction, rolling optimization, and feedback correction). The schematic diagram of the Fuzzy DMC model predictive controller for automatic train operation is shown in Figure 2.

**Figure 3:**
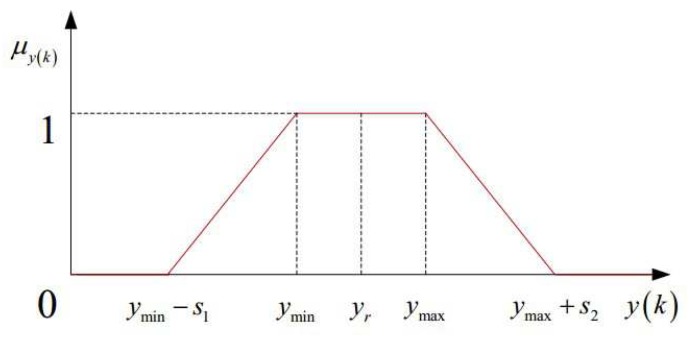
Diagram for fuzzy satisfaction degree calculation $\mu_{y\left(k\right)}$ of system output y(k).

As can be seen from Figure 3, it is impossible to obtain the actual output value (real-time velocity) for automatic train operation tracking control system. In addition, as can be seen from Formula (7), the real-time softness factor is also an important factor that impacted the multi-objective performance index for automatic train operation tracking control. Therefore, it is necessary to improve the real-time velocity sampling accuracy and softness factor accuracy for automatic train operation tracking control as much as possible.

## 3. Model Predictive Controller Based on Online Obtaining of Softness Factor and Fusion Velocity

### 3.1. Fusion Velocity Computation Model and Corrected Model Based on Online Obtaining

#### 3.1.1. Fusion Velocity Computation Model Based on Online Obtaining

According to the multi-objective performance index for automatic train operation tracking control, the fusion velocity model based on online obtaining is necessary to take into account the energy consumption, running time, comfort, and parking accuracy. Taking into account the sampling effect, hardware technology (storage and computing ability), funds, space, and other factors, three kinds of velocity sampling sources are sufficient (motor speed, motor torque and train instantaneous displacement) and are selected and synthesized. The fusion velocity computation model based on online obtaining is established as follows,

(14) vis,v=nis×trntv×ηg×ηis,T×ηis,itcvis,F=vis−1,a+Fis−wisM·Δtvis,s=ΔSΔt=sis−sis−1Δt

(15) $$\begin{array}{c} v_{is,a}=\lambda_{is,v}\times v_{is,v}+\lambda_{is,F}\times v_{is,F}+\lambda_{is,s}\times v_{is,s} \end{array}$$

where $v_{is,a}$ represents the final calculated velocity by speed analyzer ultimately of the *i*-th sampling point; $v_{is,v}$ represents the velocity calculated based on actual motor speed sampled of the *i*-th sampling point; $v_{is,F}$ represents the velocity calculated based on actual motor torque sampled of the *i*-th sampling point; $v_{is,s}$ represents the velocity calculated based on actual train instantaneous displacement sampled of the *i*-th sampling point; $n_{is}$ represents the actual motor speed sampled by speed sensors of the *i*-th sampling point; $tr_{ntv}$ represents the transmission ratio of the motor speed to train velocity; $\eta_{g}$ represents the degree of tooth engagement between the gears; $\eta_{is,T}$ represents the speed transmission efficiency of the *i*-th sampling point; $\eta_{is,itc}$ represents the efficiency for the train to overcome idling, taxiing, and creep sliding of the *i*-th sampling point; $F_{is}$ represents the force calculated based on actual motor torque sampled of the *i*-th sampling point; Fis=ηis,F×TisRmr; *T_is_* represents the actual motor torque sampled by torquemeter of the *i*-th sampling point; *η_is,F_* represents the force comprehensive transmission efficiency of the *i*-th sampling point; *R_mr_* represents the radius of motor rotor; *wis*_(*v*,*s*)_ represents the actual resistance of the *i*-th sampling point (*v*,*s*); Δ*_t_* represents the sampling time-interval, Δ*_t_* is 500 μs in this paper; Δ*_s_* represents the sampling displacement-interval; *s_is_* represents the actual train instantaneous displacement sampled by displacement pickup of the *i*-th sampling point; and *λ_is_* = {*λ_is,v_*, *λ_is,F_*, *λ_is,s_*} represents the synthetic weight of the velocity sampled by different ways.

Synthetic weight is vital for real-time sampling precision in the tracking control process, the importance of each velocity sampling sources needs to be considered, so as to give the appropriate synthetic weight. The synthetic weight indicates the importance of the real-time velocity obtained by different speed sampling sources. Yet, the selection of the synthetic weight by traditional methods lacks the specific theoretical basis, so there is certain subjective limitation in actual applied. As automatic train operation tracking control is an extremely complex issue, there is a trajectory characteristic for automatic train operation tracking control curve dominated by velocity target curve, train parameters, line conditions and running requirements, and the traditional methods for setting synthetic weight based on experience empower is subjective and blind, so it is necessary to be improved. In this paper, an synthetic weight empower using entropy method is applied for automatic train operation tracking control. First, the whole tracking control curve is divided by a position according to trajectory characteristic and line conditions; second, a large number of real-time data, including velocity, force, and position information for the whole tracking control process, should be sampled to prepare for calculation; finally, the entropy method is used to calculate the synthetic weight of each divided subinterval of tracking control curve.

Entropy is a measure of uncertainty for information calculation. The entropy weight method is utilizes the entropy characteristics and assigns a weight to each index in an event by calculating the entropy value. The entropy weight method is an objective weight empower method, because it simply depends on the discreteness of data itself. The specific steps of computational process for entropy weight method are as follows.

A certain number of samples (as many as possible) must be collected to prepare for the calculation, and their index values also needed to be recorded.

To eliminate the negative influences caused by the difference between units and magnitude orders, the index values must be normalized. The calculation formulas for the normalization can be expressed as follows,

(16) $$\begin{array}{c} {x_{ij}}^{\prime }=\frac{x_{ij}-\min\left\{x_{ij},x_{2j},\ldots ,x_{nj}\right\}}{\max\left\{x_{ij},x_{2j},\ldots ,x_{nj}\right\}-\min\left\{x_{ij},x_{2j},\ldots ,x_{nj}\right\}} \end{array}$$

(17) $$\begin{array}{c} {x_{ij}}^{\prime }=\frac{\max\left\{x_{ij},x_{2j},\ldots ,x_{nj}\right\}-x_{ij}}{\max\left\{x_{ij},x_{2j},\ldots ,x_{nj}\right\}-\min\left\{x_{ij},x_{2j},\ldots ,x_{nj}\right\}}\; \end{array}$$

where $j=\left(1,2,\cdot \cdot \cdot ,m\right)$,$i=\left(1,2,\cdot \cdot \cdot ,n\right)$; *m* represents index number; *n* represents the number of samples; ${x_{ij}}^{\prime }$ represents the *j*-th processed index value of the *i*-th sample after normalization; $x_{ij}$ represents the *j*-th original index value of the *i*-th sample before normalization; max and min, respectively, represent the maximum and minimum values of the array. If index value $x_{ij}$ is a positive number, Formula (16) is used to normalize; otherwise, Formula (17) is used to normalize.

The normalized index values are necessary to filtered out the zero value further, so as to avoid illegal logarithmic function (ln(0)) in next subsequent calculation processes. The specific formula for filtered out zero value is as follows,

(18) $$\begin{array}{c} {x_{ij}}^{\prime \prime }=\Delta z+\left(1-2\times \Delta z\right)\times {x_{ij}}^{\prime } \end{array}$$

where ${x_{ij}}^{\prime \prime }$ represents the *j*-th processed index value of *i*-th sample after filtering out the zero value; Δz represents the tiny value reasonable; Δz is 0.01 in this paper.

Then, the entropy values of each index values are necessary to be calculated. The specific formulas for calculating the entropy values are as follows,

(19) $$\begin{array}{c} p_{ij}=\frac{{x_{ij}}^{\prime \prime }}{\overset{n}{\underset{i=1}{\sum }}{x_{ij}}^{\prime \prime }} \end{array}$$

(20) $$\begin{array}{c} e_{j}=-k\times \overset{n}{\underset{i=1}{\sum }}\left(p_{ij}\times \ln\left(p_{ij}\right)\right) \end{array}$$

where $p_{ij}$ represents the *j*-th index weight value of *i*-th sample in *j*-th index; $e_{j}$ represents the *j*-th index entropy value; *k* represents the entropy coefficient, the value is the reciprocal of $\ln\left(n\right)$, k=1lnn.

Finally, the index weight value could be calculated. The specific formula for calculating the index weight values is as follows,

(21) $$\begin{array}{c} d_{j}=1-e_{j} \end{array}$$

(22) $$\begin{array}{c} \lambda_{j}=\frac{d_{j}}{\overset{m}{\underset{j=1}{\sum }}d_{j}} \end{array}$$

where $d_{j}$ represents the *j*-th entropy redundancy value of *j*-th index, it indicates the difference degree of this index; $e_{j}$ represents the *j*-th index entropy value; $\lambda_{j}$ represents the *j*-th weight value.

#### 3.1.2. Fusion Velocity Corrected Model Based on Online Obtaining

If the factor of the velocity sampling sources sampled inaccurately is not considered, the improvement effect for automatic train operation tracking control will inevitably be restricted. To improve the real-time sampling velocity precision, a corrected method of real-time sampling velocity for automatic train operation tracking control using auxiliary corrective velocity sampling source is popularly applied in various types of urban rail vehicles. The specific evaluation and corrected formulas are as follows,

(23) $$\begin{array}{c} \Delta v_{is,c}=\left|v_{is,x}-v_{is,ref}\right|\leq \Delta v_{is,p} \end{array}$$

(24) $$\begin{array}{c} v_{is,c}=\frac{\Delta v_{is,p}}{\Delta v_{is,c}}\times v_{is,x}+\frac{\Delta v_{is,c}-\Delta v_{is,p}}{\Delta v_{is,c}}\times v_{is,ref} \end{array}$$

where $v_{is,x}$ represents a velocity sampling source *x* from $v_{is,v},v_{is,F},v_{is,s}$ using to synthetic final calculated velocity $v_{is,a}$; $v_{is,ref}$ represents the reference velocity (auxiliary corrective velocity sampling source) based on actual sampling data sampled by specific auxiliary sensor; $\Delta v_{is,c}$ represents the actual error value between $v_{is,x}$ and $v_{is,ref}$; $\Delta v_{is,p}$ represents the maximum permit satisfied error value between $v_{is,x}$ and $v_{is,ref}$; $v_{is,c}$ represents the final corrected value calculated by Formula (24) when correctness condition (Formula (23)) is not satisfied.

Reference velocity $v_{is,ref}$ will exert a measure of influence over the velocity-corrected effect. Thus, the choice of auxiliary corrective velocity sampling source is significant. The specific gear speed on the vehicle wheel side of the gear box is a good choice.

### 3.2. Softness Factor Adaptive Adjusting Model Based on Online Obtaining

The softness factor is a key parameter for DMC MPC; it plays plays an important role in balancing the degree of robustness and rapidity for the DMC MPC tracking control system. If softness factor α is chosen as a larger value, the system will have slower response speed and stronger robustness; by contrast, if softness factor α is chosen as a smaller value, the system will have faster response speed and worse robustness [36]. Thus, both response speed and robustness must be taken into account for softness factor α setting.

Considering the trajectory characteristic and tracking control condition for automatic train operation, the softness factor adaptive adjusting model based on online obtaining is established as follows,

(25) $$\begin{array}{c} \alpha =\lambda_{\alpha Ts}\times \alpha_{Ts}\left(s\right)+\lambda_{\alpha_{\mu y}}\times \alpha_{\mu y}\left(y\left(k\right),y_{r}\right) \end{array}$$

where α represents the final calculated real-time softness factor; $\alpha_{Ts}$ represents the real-time softness factor calculated based on the trajectory characteristic of the present position; $\alpha_{\mu y}\left(y\left(k\right),y_{r}\right)$ represents the real-time softness factor calculated based on tracking control condition of the present system output $y\left(k\right)$; $\lambda_{\alpha Ts}$ and $\lambda_{\alpha_{\mu y}}$ represent the fusion weights of $\alpha_{Ts}$ and $\alpha_{\mu y}\left(y\left(k\right),y_{r}\right)$, respectively; and $\lambda_{\alpha Ts}+\lambda_{\alpha_{\mu y}}=1$.

The whole tracking control curve must be divided into several different types of subintervals by position according to the trajectory characteristic and line conditions. The specific types of subintervals are described as follows.

Type 1: The vibrating area nearby inflection point of tracking control curve.

In this area, there is the strong velocity fluctuation in the velocity trajectory. Thus, aiming at improving the system robustness as much as possible, softness factor $\alpha_{Ts}$ should be an appropriate larger value at the cost of reduce acceptable system rapidity.

Type 2: The smooth area of tracking control curve.

In this area, there is no obvious velocity fluctuation in the velocity trajectory. Thus, aiming at improving the system rapidity as much as possible, softness factor $\alpha_{Ts}$ should be chosen as an appropriate smaller value at the cost of reduce acceptable system robustness.

Type 3: The connected area in the middle of smooth area and the vibrating area of the tracking control curve.

In this area, the system rapidity and rapidity are taken into account for softness factor $\alpha_{Ts}$ setting. Thus, softness factor $\alpha_{Ts}$ should be choose a appropriate intermediate value.

In addition, although in the same type of subintervals, the softening factor $\alpha_{Ts}$ almost varies because of the different intensity degrees of velocity fluctuation. The specific calculation formula for softness factor $\alpha_{Ts}$ based on trajectory characteristic of the present position is described as follows,

(26) $$\begin{array}{c} \alpha_{Ts}\left(s\right)=\left\{\begin{array}{c} \alpha_{Tr,si}+\left(\alpha_{Tr,si}-\alpha_{Tr,si-1}\right)\times \frac{\left(\left(S_{si}+S_{1}\right)-s\right)}{S_{1}+S_{2}}\;\;\;\;\;\;s<S_{si}+S_{1}\\ \alpha_{Tr,si}\;\;\;\;\;\;\;\;\;\;\;\;\;\;\;\;\;\;\;\;\;\;\;\;\;\;\;\;\;\;\;\;\;\;\;\;\;\;\;\;\;\;\;\;\;S_{si}+S_{1}\leq s\leq S_{si+1}-S_{2}\\ \alpha_{Tr,si}+\left(\alpha_{Tr,si+1}-\alpha_{Tr,si}\right)\times \frac{\left(s-\left(S_{si}-S_{2}\right)\right)}{S_{1}+S_{2}}\;\;\;\;\;s>S_{si+1}-S_{2} \end{array}\right. \end{array}$$

where si represents the subinterval index $si\in \left\{1,2,\ldots ,si_{max}\right\}$; si_max represents the number of subintervals; $S_{si}$ represents the starting position of the si-th subinterval; $S_{si\_max+1}$ represents the terminal position of tracking control curve, it is a target parking position; $\alpha_{Tr,si}$ represents the reference value of soften factor in the si-th subinterval; $\alpha_{Ts,0}=\alpha_{Ts,1}$, $\alpha_{Ts,si\_\max+1}=\alpha_{Ts,si\_\max}$; $S_{1}$ and $S_{2}$ represent the connected length, in the connected area; the softness factor $\alpha_{Ts}$ is reduced or increased linearly and smoothly, so as to avoid the instability of tracking control system.

Aiming at solving this control problem with fuzzy characteristic, an fuzzy adaptive adjusting method for online obtaining softness factor $\alpha_{\mu y}$ is applied. First of all, the satisfaction degree of control is defined, so as to the automatic train operation tracking control problem can be transformed into an optimization decision-making problem by fuzzy reasoning; then, the corresponding real-time parameters of the controller are adjusted online to meet the requirements of the system control quality, so as to achieve the purpose of system optimization control. The specific calculation formula for fuzzy satisfaction degree $\mu_{y\left(k\right)}$ of system output y(k) is as follows,

(27) $$\begin{array}{c} \mu_{y\left(k\right)}=\left\{\begin{array}{ccccc} 0 & & & & y\left(k\right)<y_{\min}-s_{1}\\ 1+\frac{y\left(k\right)-y_{\min}}{s_{1}} & & & & y_{\min}-s_{1}\leq y\left(k\right)<y_{\min}\\ 1 & & & & y_{\min}\leq y\left(k\right)<y_{\max}\\ 1+\frac{y\left(k\right)-y_{\max}}{s_{2}} & & & & y_{\max}\leq y\left(k\right)<y_{\max}+s_{2}\\ 0 & & & & y_{\min}-s_{1}\geq y\left(k\right) \end{array}\right. \end{array}$$

where $s_{1}$ and $s_{2}$ represent the blur width, which can indicate the requirement of designer, if $s_{1}=s_{2}=0$, the requirements for the control system are strict, and the automatic train operation tracking control is not so, and this represents a combination of the practical situation; $y_{\max}$ and $y_{\min}$ represent the maximum and minimum value of design expectation, respectively, if $y_{\max}=y_{\min}$, it will be shown as trigonometric membership function; otherwise, it will be shown as trapezoid membership function. The corresponding diagram for fuzzy satisfaction degree calculation $\mu_{y\left(k\right)}$ of system output y(k) is shown in Figure 3.

The error between the output value and the reference target value of system (i.e., fuzzy satisfaction degree $\mu_{y\left(k\right)}$ of system output y(k)) should also be considered. If the fuzzy satisfaction degree $\mu_{y\left(k\right)}$ is larger, it can indicate that the error between the output value and the reference target value of system is smaller, at this time, there is a small overshoot of the system and softness factor $\alpha_{\mu y}$ so that an appropriate lager value needs to chosen to increase the system rapidity; by contrast, there is an obvious overshoot of the system and softness factor $\alpha_{\mu y}$ so that an appropriate small value needs to be chosen to reduce the system rapidity to ensure system robustness [36]. According to the influence of softness factor $\alpha_{\mu y}$ for the system dynamic response, the specific exponential calculation formula for softness factor $\alpha_{\mu y}$ by fuzzy satisfaction degree $\mu_{y\left(k\right)}$ of system output y(k) is as follows,

(28) $$\begin{array}{c} \alpha_{\mu y}\left(y\left(k\right),y_{r}\right)=\alpha_{\max}+\left(\alpha_{\max}\times e^{-\left(b\times \mu_{y\left(k\right)}\right)}-\alpha_{\max}\right)\times \mu_{y\left(k\right)} \end{array}$$

where $\alpha_{\max}$ represents the maximum value of softness factor $\mu_{y\left(k\right)}$; *b* represents the gain coefficient; it determines the shape of the softening factor $\alpha_{\mu y}\left(y\left(k\right),y_{r}\right)$ function curve.

**Figure 4:**
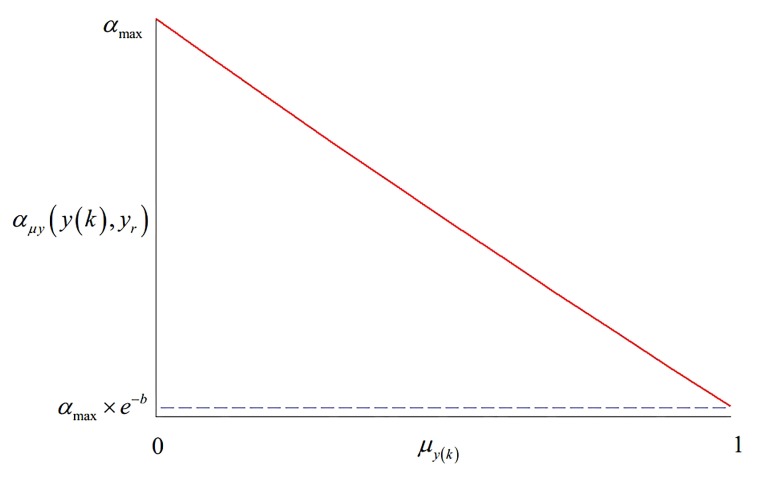
Diagram for fuzzy satisfaction degree calculation $\mu_{y\left(k\right)}$ of fuzzy satisfaction degree calculation $\mu_{y\left(k\right)}$.

**Figure 5:**
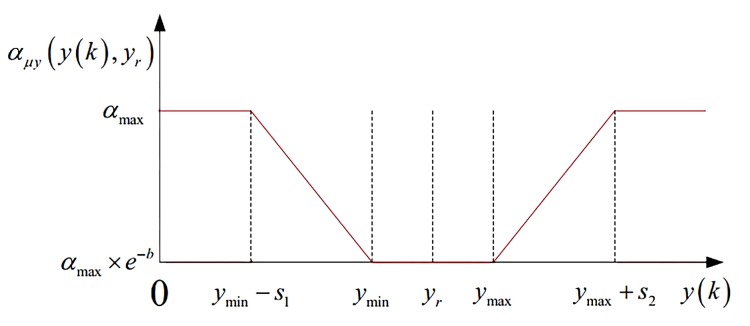
Diagram for fuzzy satisfaction degree calculation $\mu_{y\left(k\right)}$ of system output y(k).

The corresponding diagram for softness factor $\alpha_{\mu y}$ of the fuzzy satisfaction degree calculation $\mu_{y\left(k\right)}$, and softness factor $\alpha_{\mu y}$ of system output y(k) are shown in Figure 4 and Figure 5.

### 3.3. Improved Whale Optimization Algorithm for Softness Factor Adaptive Adjusting Parameters Optimization

Optimization algorithms are used to obtain a set of adjustable parameters for the satisfactory tracking control effect in actual automatic train operation scenarios. The specific softness factor adaptive adjusting parameters optimization model is as follows,

(29) $$\begin{array}{c} \begin{array}{ccccc} \min & & & & F\left(x\right)=\left(P_{k},\frac{ITAE}{\max\left(ITAE\right)},\frac{K_{safe}}{\max\left(K_{safe}\right)}\right)\\ s.t. & & & & x=(\lambda_{\alpha Ts},\lambda_{\alpha \mu y},\alpha_{Tr},\alpha_{\max},b)\\ & & & & g_{ig}\left(x\right)\leq 0,\;ig=1,2,\cdots ,ng\\ & & & & x\in \Omega^{\prime \prime } \end{array} \end{array}$$

where *x* F(x) represents the target vector; $\Omega^{\prime \prime }$ represents feasible solution space of *x*; $g_{ig}\left(x\right)$ represents the *ig*-th equality or inequality constraint for automatic train operation tracking control problem, ng represents the number of equalities and inequality constraints; the five adjustable parameters ($\lambda_{\alpha Ts}$, $\lambda_{\alpha \mu y}$, $\alpha_{Tr}$, $\alpha_{\max}$, *b*) are decision variables.

Objective decomposition is an effective method to solve the multi-objective optimization problems. The Tchebycheff decomposition method is selected in this paper among many objective decomposition methods [37]. The specific calculation formula for the aggregate function value of the Tchebycheff decomposition method is as follows,

(30) $$\begin{array}{c} \begin{array}{c} m\mathrm{in}g^{te}\left(x|\lambda ,z^{*}\right)=\max_{1\leq i\leq m}\{\lambda_{i}|f_{i}\left(x\right)-z_{i}^{*}\left|\right\}\\ s.t.x\in \Omega^{\prime \prime } \end{array} \end{array}$$

where $z^{*}$ represents the reference point, $(z_{i}^{*}=\min\left\{f_{i}\left(x\right)|x\in \Omega \right\}$, i=1,…,m), which is the optimal solution of each objective function at present; $\lambda_{i}$ is the weight of the *i* th objective, $\overset{m}{\underset{i=1}{\sum }}\lambda_{i}=1$.

Whale optimization algorithm with strong global optimization ability is chosen in this paper. Whale optimization algorithm (WOA) is a new metaheuristic optimization algorithm learned from whale predatory behavior. There are two operators (position update and prey searching) in the computation process of the whale optimization algorithm [38]. The specific calculation formula for the position update of the basic whale optimization algorithm is as follows,

(31) $$\begin{array}{c} X\left(t+1\right)=\left\{\begin{array}{c} X^{*}\left(t\right)-A\cdot D\begin{array}{cc} & p<P_{s} \end{array}\\ X^{*}\left(t\right)+D_{p}\cdot e^{Bl}\cdot \cos\left(2\pi l\right)\begin{array}{cc} & p\geq P_{s} \end{array} \end{array}\right. \end{array}$$

where $X^{*}\left(t\right)$ represents the optimal position vector obtained by the current optimization; $D_{p}=\left|X^{*}\left(t\right)-X\left(t\right)\right|$ represents the distance between humpback whales and their prey; *p* represents the behavioral selection probability of humpback whales, $p\in \left[0,1\right]$; $P_{s}$ represents the probability of surrounding prey of humpback whales, $P_{s}\in \left[0,1\right]$; the probability of spiral hunting is $1-p_{s}$; *B* represents a constant, which is used to define the shape of spiral; *l* represents the random number in (−1,1); *t* is the current iteration number; $T_{max}$ is the maximum number of iterations; *a* represents convergence factor; *A* and *C* represent the correlation coefficients respectively; $r_{1}$ and $r_{2}$ are random numbers, $r_{1}\in \left[0,1\right]$, $r_{2}\in \left[0,1\right]$.

The specific calculation formulas for convergence factor *a*, correlation coefficients *A*, and *C* is as follows,

(32) $$\begin{array}{c} a=2-2\times t/T_{\max} \end{array}$$

(33) $$\begin{array}{c} A=2a\times r_{1}-a \end{array}$$

(34) $$\begin{array}{c} C=2\times r_{2} \end{array}$$

After the position updated, prey searching is implemented by means of random individual positions. The specific calculation formula for prey searching of the basic whale optimization algorithm is as follows,

(35) $$\begin{array}{c} D=\left|CX_{rand}-X\left(t\right)\right| \end{array}$$

(36) $$\begin{array}{c} X\left(t+1\right)=X_{rand}-A\cdot D \end{array}$$

where $X_{rand}$ is the position vector of randomly selected whales. If A≥1, a search leader individual is randomly selected, and the position of other whales is updated based on the whale position of the leader individual, so as to guide the whales to leave the prey and find a more suitable prey to enhance the global search ability of the algorithm.

The relatively fixed method of linear decline of convergence factor *a* will reduce the population diversity maintenance ability, so that the algorithm can easy to fall into local convergence in the late iteration. Aiming at solving this problem, the strategy of cosine decline combined with chaotic random method for convergence factor nonlinear decline is proposed in this paper. The specific calculation formula is as follows,

(37) $$\begin{array}{c} \begin{array}{c} a=2\cdot \cos\left(\frac{\pi }{2}\cdot \frac{t}{T_{\max}}\right)\begin{array}{cc} & p_{a}\left(t\right)<P_{a} \end{array}\\ a=2\cdot rand^{2}\cdot \sin\left(\pi \cdot rand\right)\begin{array}{cc} & p_{a}\left(t\right)>P_{a} \end{array} \end{array} \end{array}$$

where $p_{a}\left(t\right)$ represents the behavioral selection probability of the convergence factor *a*, $p_{a}\left(t\right)\in \left[0,1\right]$; $P_{a}$ represents the probability of cosine decline of the convergence factor *a*, $P_{a}\in \left[0,1\right]$; and the probability of chaotic random is $1-P_{a}$.

Compared with the linear decline strategy, the decline rate of the convergence factor is significantly different in the whole iteration cycle caused by the nonlinear decline strategy with a certain degree of chaos uncertainty for convergence factor *a*, and it is helpful for maintaining the population diversity, thus the algorithm global convergence performance will be improved [39].

According to the Tchebycheff decomposition method, the aggregate function value is the fitness index for the multi-objective optimization algorithm. After the computation process of each iteration, the newly generated non-dominated solutions of the current population are put into the elite archive. The archive must kept within a certain size by some elite individuals with small differences from other elite individuals, so as to avoid computational burden of the algorithm. According to the updating rules of the whale optimization algorithm, the reference point $z^{*}$ plays an important role in guiding the direction of global convergence, and a certain degree of local convergence due to this fixed foraging behavior. Meanwhile, the selector, crossover, and mutation of the genetic algorithm can generate a large number of new solutions with great differences for the whale optimization algorithm based on evolutionary processes, so as to further improve the global convergence performance due to more powerful population diversity maintenance ability.

The specific steps of improved whale optimization algorithm proposed in this paper are as follows.

Step 1: Initialization.

Initialize the whale population (the size is Nw), and the Tchebycheff aggregation function values of each whale individual are calculated.

Step 2: Iterative computations.

the Archive is obtained;

Archive=∅, the reference point $z^{*}=\left(z_{1}^{*},z_{2}^{*},\ldots ,z_{m}^{*}\right)$, and $z_{j}^{*}=\min\left(f_{j}\left(x\right)\right)$, j=1,2,…,m, *m* represents the number of objectives, a uniformly distributed weight vector set $\lambda_{0}$ is generated, and $\lambda^{1}=\lambda_{0}$.

If the current iteration number is greater than 1, the weight $\lambda^{t,i}$ for solution $x^{t,i}$ are need to be recalculated. According to the literature [40], in the *t*-th iteration, the specific calculation formula for weight $\lambda^{t,i,k}$ of the *k*-th optimization index of the *i*-th individual (solution) $x^{t,i}$ in the population is as follows,

(38) $$\begin{array}{c} \lambda^{t,i,k}=\frac{1}{f{\left(x^{t,i}\right)}^{k}-Z^{ref,k}}{\left(\overset{m}{\underset{ik=1}{\sum }}\frac{1}{f{\left(x^{t,i}\right)}^{ik}-Z^{ref,ik}}\right)}^{-1} \end{array}$$

where $i\in \left\{1,2,\ldots ,Nw\right\}$, $k\in \left\{1,2,\ldots ,m\right\}$.

For any solution target $z^{c}=\left(z_{1}^{c},z_{2}^{c},\ldots ,z_{m}^{c}\right)$ of Pareto front, its weight vector is $\frac{1}{f\left(x\right)-Z^{c}}{\left(\overset{m}{\underset{ik=1}{\sum }}\frac{1}{f\left(x\right)-Z^{c,ik}}\right)}^{-1}$. Because the Pareto front is not easily available, it is replaced by the nearest solution target $Z^{ref}$ in Archive;

The strategy of cosine decline combined with chaotic random method is used to calculate convergence factor *a*;

The updating rules of the whale optimization algorithm are used to update each individual whale.

Step 3: The archive and genetic evolution mechanism.

The Pareto front of the current whale population is obtained, and it is used to expand the Archive.

Some elite individuals with small differences from other elite individuals are deleted, until the size of Archive is not exceed allowed limit archive size NA.

Three operators (selection, crossover, and mutation) of the genetic algorithm are applied in the whole whale population, so as to further improve the population diversity maintenance ability.

Step 4: Termination judging.

The hypervolume indicator is chosen as termination judging indicator. Using the hypervolume indicator of the dominated portion of the objective space as a measure for the quality of Pareto set approximations is appropriate and effective. The specific formula for hypervolume indicator for objective vector set *A* with reference point (0,0,...,0) is as follows.

(39) $$\begin{array}{c} {I^{*}}_{H}\left(A\right)=\int_{\left(0,0,\ldots ,0\right)}^{\left(1,1,\ldots ,1\right)}\alpha_{A}\left(z\right)dz \end{array}$$

where *A* is any objective vector set in objective space Ω; if there is an objective vector *a*, Pareto superior *z* and *a* belongs to *A*, $\alpha_{A}\left(z\right)=1$; otherwise, $\alpha_{A}\left(z\right)=0$ [41].

Thus, the hypervolume indicator indicates the dominated portion in objective space Ω. Generally, Hypervolume as Klee’s Measure Problem (HKMP) is an effective calculation method for hypervolume indicator [42]. As only 3 objects $\left(P_{k},\frac{ITAE}{\max\left(ITAE\right)},\frac{K_{safe}}{\max\left(K_{safe}\right)}\right)$ must be taken into account, the calculation method by using equivalent volume model for the volume of irregular objects can be used. The specific formulas for the hypervolume indicator for 3 objects by using equivalent volume model is as follows,

(40) $$\begin{array}{c} {I^{*}}_{H}\left(A\right)=\frac{\overset{na}{\underset{ia=1}{\sum }}\overset{nb}{\underset{ib=1}{\sum }}\overset{nc}{\underset{ic=1}{\sum }}\alpha_{A}\left(cp(ia,ib,ic)\right)}{na\times nb\times nc} \end{array}$$

where na, nb, and nc are the split numbers for each normalization objective domain (0,1); cp(ia,ib,ic) represents the central point of the (ia,ib,ic)th cube of normalization objective space.

**Figure 6:**
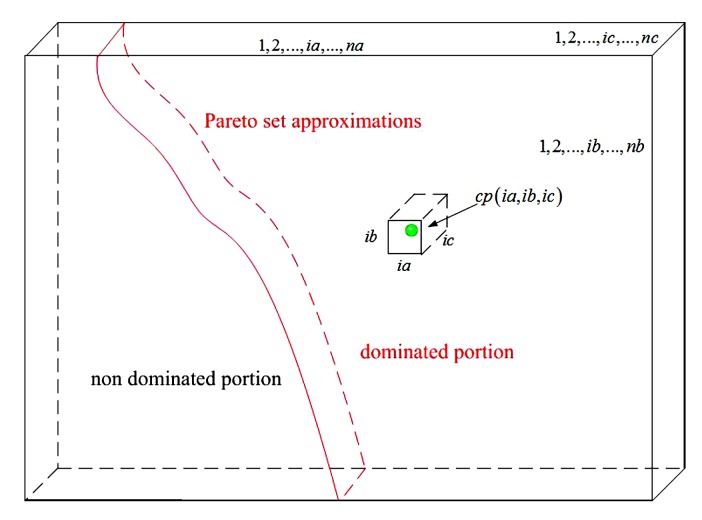
Schematic diagram of hypervolume indicator for 3 objects by using equivalent volume model.

The schematic diagram of the hypervolume indicator for 3 objects by using equivalent volume model is shown in Figure 6.

If the hypervolume indicator ${I^{*}}_{H}\left(A\right)$ is reached beforehand, an unchanged number of hypervolume indicators nH will be resetted; otherwise, make nH=nH+1.

If the maximum unchanged number of the hypervolume indicator nHmax is reached, the calculation will be terminated; otherwise, return Step 2.

**Figure 7:**
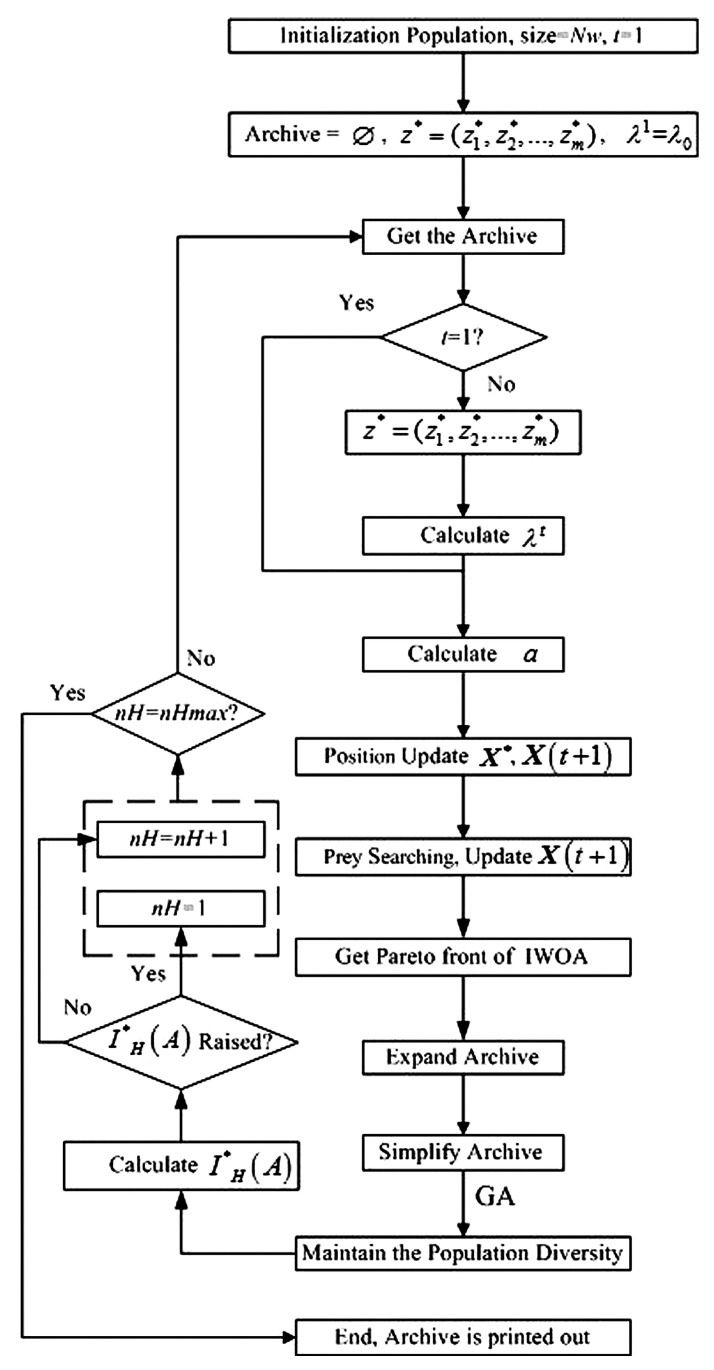
The flowchart of the improved whale optimization algorithm proposed in this paper.

The flowchart of improved whale optimization algorithm proposed in this paper is shown in Figure 7.

Aiming at improving the global searching ability, the evolution law is must be considered in the computation process. The excellent individuals should have a small mutation probability, so that they can accumulate optimization results effectively, and the poor individuals should choose a large mutation probability, which can be fully eliminated, so as to enhance the capacity of exploration [43]. The mutation probability calculation formula based on sigmoid function $y=\frac{1}{1+e^{-x}}$ is as follows,

(41) $$\begin{array}{c} p_{m}=p_{m\_\max}\frac{1}{1+e^{-ap\left(N_{s}-fit{\left(x\right)}^{\prime }\right)}} \end{array}$$

where $P_{m}$ is the mutation probability value; $p_{m\_\max}$ is the maximum mutation probability; ap is the shape factor for the sigmoid function of mutation probability; $N_{s}$ is the demarcation point of the whale population; $fit{\left(x\right)}^{\prime }$ is the normalized value of fitness function value $fit\left(x\right)$ of whale individual *x* in the whale population.

This mutation probability calculation method has certain fairness, and the whale individuals have appropriate mutation probability according to fitness function value, so as to prevent the population controlled by advantage individuals and persist evolution opportunity for disadvantaged individuals.

A multimodal crossover method is conducive to finding a more satisfactory optimal solution for the complex optimization issue [44,45]. The multimodal crossover combining popular blended crossover and unimodal normal distribution crossover is applied in this paper. The specific calculation formula for multimodal crossover is as follows,

(42) $$\begin{array}{c} \begin{array}{c} x_{c}=r_{p1}\times x_{p1}+\left(1-r_{p1}\right)\times x_{p2}\;\;\;\;\;\;\;\;\;\;\;\;\;pc<pc_{b}\\ x_{c}=x_{p}+\varepsilon d+\overset{\mu }{\underset{ic=1,ic\neq p}{\sum }}\nu_{ic}e_{ic}\;\;\;\;\;\;\;\;\;\;\;\;\;pc_{b}<pc<\left(pc_{b}+pc_{u}\right) \end{array} \end{array}$$

where $x_{c}$ is the solution after multimodal crossover operation; $x_{p1}$ and $x_{p2}$ are two parent solutions for blended crossover; pc is the behavioral selection probability about multimodal crossover operation; $pc_{b}$ and $pc_{u}$ are the crossover probabilities for blended crossover and unimodal normal distribution crossover, respectively; $x_{p}$ is the midpoint for μ parent solutions for unimodal normal distribution crossover; *d* is the differential vector; $e_{ic}$ is the ic th orthogonal basis; ε and $\nu_{ic}$ are the random numbers obey normal distribution $N\left(0,{\sigma_{1}}^{2}\right)$ and $N\left(0,{\sigma_{2}}^{2}\right)$.

### 3.4. Performance Analysis of Optimization Algorithms Based on Standard Test Functions

**Figure 8:**
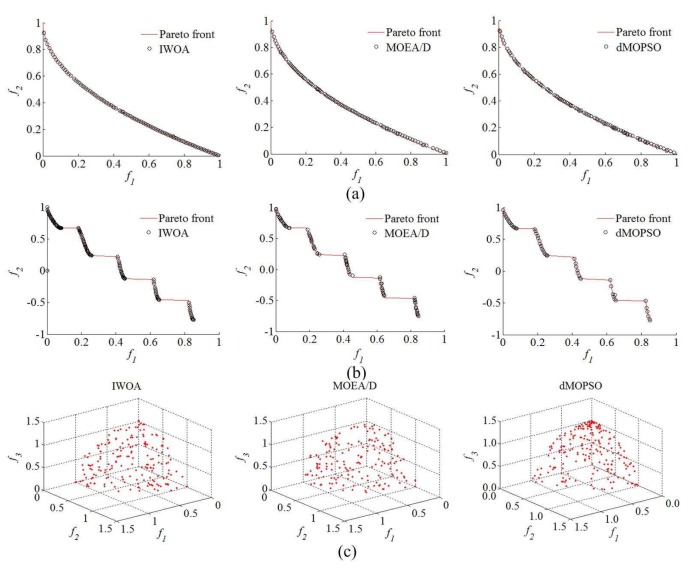
The optimization results for test functions of each optimization algorithms. (**a**) Optimization results for ZDT1. (**b**) Optimization results for ZDT3. (**c**) Optimization results for DTLZ2.

Aiming at verifying the effectiveness of IWOA proposed in this paper, standard test functions (ZDT1, ZDT3, and DTLZ2) are selected as optimization objects, and multi-objective particle swarm optimization based on decomposition (dMOPSO) [46] and multi-objective evolutionary algorithm based on decomposition (MOEA/D) [47] are selected as contrasted optimization algorithms. The improved whale optimization algorithm parameters are set as follows; maximum number of iterations is 100; probability of surrounding prey of humpback whales $P_{s}$ is 0.6; population size Nw is 50; allowed limit archive size NA is 30; probability of cosine decline of the convergence factor $P_{a}$ is 0.9; shape constant of spiral *b* is 1; shape factor for sigmoid function of mutation probability ap is 4.9; demarcation point of whale population $N_{s}$ is 0.8; probability of blended crossover $pc_{b}$ and unimodal normal distribution crossover $pc_{u}$ are 0.3 and 0.3, respectively; selection probability is 0.5; split number for each normalization objective na, nb, and nc are all 50; the maximum unchanged number of hypervolume indicator nHmax is 25. The Matlab/simulink platform is used for verifying, and the Matlab/simulink revision and the computer processor type are 2016b, MathWorks and CPU Core i9-7920X @ 2.9GHZ. The specific optimization results (approximate Pareto solution set) for test functions of each optimization algorithms are shown in Table 1 and Table 2 and Figure 8.

**Figure 9:**
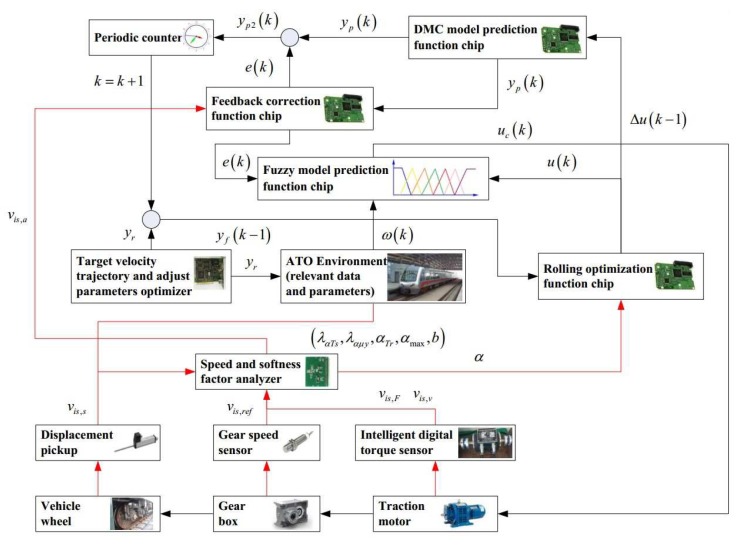
The schematic diagram of improved DMC model predictive controller for automatic train operation designed in this paper.

As can be seen from Table 1 and Table 2, compared with other optimization algorithms (dMOPSO and MOEA/D), IWOA has been improved to a considerable extent, not only in the computation speed, but also in the optimization effect for approximate Pareto solution set reflected by the hypervolume indicator ratio $\frac{{I^{*}}_{H}\left(A\left(op\right)\right)}{{I^{*}}_{H}\left(A\left(tp\right)\right)}$, $A\left(op\right)$ and $A\left(tp\right)$ are the approximate Pareto solution sets obtained by optimization algorithms and real Pareto front set, respectively. According to Figure 9, compared with other optimization algorithms (dMOPSO and MOEA/D), only the better approximate Pareto solution sets closer to the Pareto fronts of ZDT1, ZDT3, and DTLZ2 were found using IWOA, but also the distribution for obtained approximate Pareto solution set was more evenly. This indicates that the IWOA proposed in this paper has better optimization effectiveness.

### 3.5. Improved DMC Model Predictive Controller and Hardware-In-The-Loop Simulation Platform

Based on the traditional Fuzzy DMC model predictive controller, a new functional module is necessary to be realized the function of online obtaining of softness factor and fusion velocity. The schematic diagram of improved DMC model predictive controller for automatic train operation designed in this paper is shown in Figure 9.

**Figure 10:**
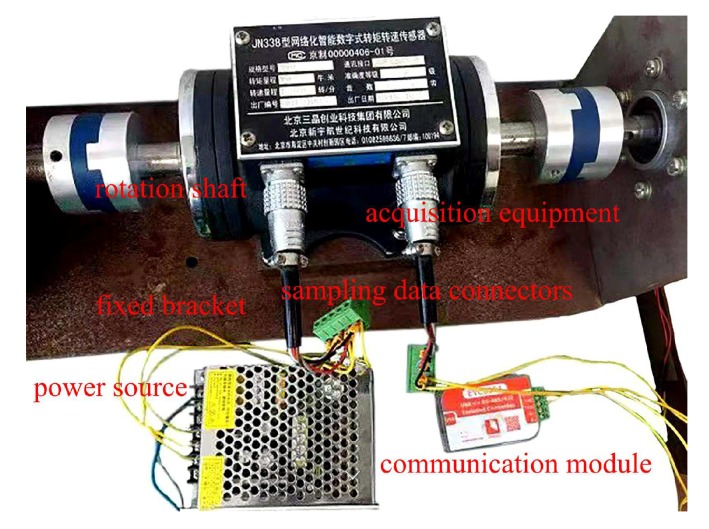
The physical diagram of the intelligent digital torque sensor.

In Figure 9, the improved DMC model predictive controller could provide control commands for the corresponding equipments in real-time using fuzzy DMC MPC based on online obtaining of softness factor and fusion velocity, enabling the the urban rail vehicle to track the target velocity trajectory; the Speed and softness factor analyzer could provide the precision instantaneous fusion velocity $v_{is,a}$ and softness factor α for rolling optimization and feedback correction based on three kinds of velocity sampling sources ($v_{is,v}$, $v_{is,F}$, and $v_{is,s}$) and a set of softness factor α adjustable parameters. The intelligent digital torque sensor, gear speed sensor, and displacement pickup are data acquisition devices. The physical diagram of the intelligent digital torque sensor is shown in Figure 10.

In Figure 10, the acquisition equipment is an intelligent digital torque sensor of model No. JN338; the fixed bracket is made of iron and has two functions of fixing and preventing jitter; the power source supplies electricity to torque sensor of model No. JN338; the communication module transmits the real-time value of motor speed and torque using the 485 communication protocol; the sampling data connectors are connectors for sampling data (motor speed and torque); and can be connected to communication module, controller, or other equipment; rotation shaft of intelligent digital torque sensor must be connected to rotation shaft of traction motor and load (breaker or rheostat box).

**Figure 11:**
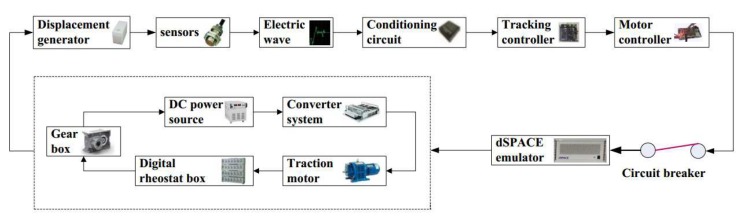
The structure diagram of the hardware-in-the-loop simulation (HILS) platform used in this paper for automatic train operation tracking control scenario.

**Figure 12:**
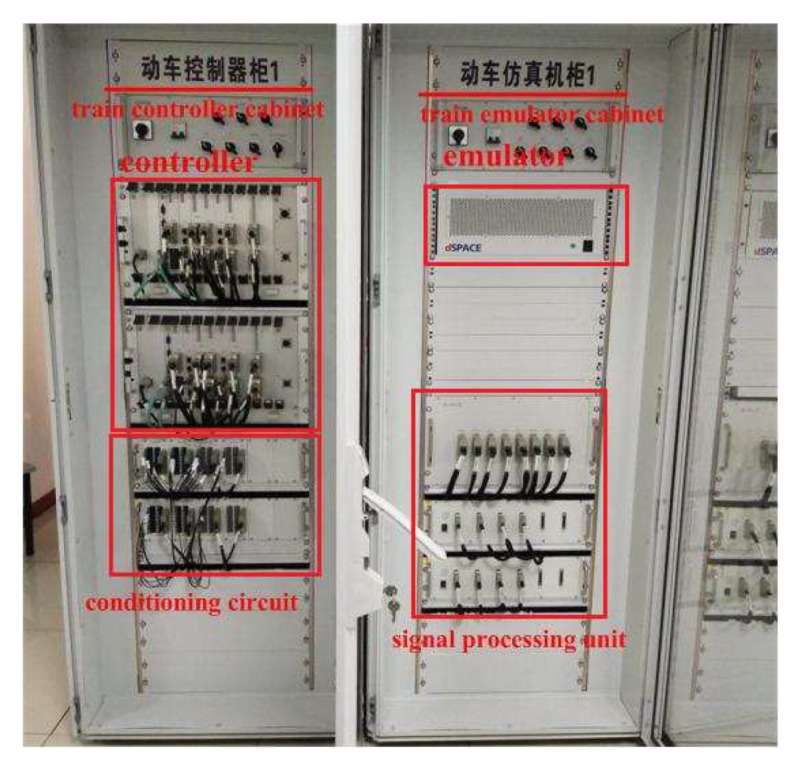
The physical diagram of controller cabinet and simulation cabinet for HILS platform.

To more effectively test the performance of the tracking control algorithm in actual automatic train operation tracking control scenarios, the dSPACE HILS technology is adopted. In this way, the optimization algorithm or control algorithm needed to be verified is written into the chip of the optimizer or controller. The structure diagram of HILS platform used in this paper for automatic train operation tracking control scenario, and the physical diagram of controller cabinet and simulation cabinet for HILS platform are shown in Figure 11 and Figure 12.

In Figure 11, the Displacement generator is used to generate the train instantaneous displacement, so that the static (no actual displacement) HILS platform can truly reflect the actual automatic train operation tracking control scenario; various sensors are used to feed electrical waves of sampling sources back to the Controller in real-time; the Conditioning circuit can regulate electrical signals properly for the Tracking controller appropriately; the Motor controller could provide electrical control commands for Traction moto and other corresponding equipments of Electrical loop in real-time using a proper electrical control algorithm. DC power source, Converter system, Traction motor, Digital rheostat box, and Gear box are simulation electric hardware equipments.

In Figure 12, the ’train controller cabinet’ and ’train emulator cabinet’ are the vital equipments for automatic train operation HILS, except for the controller and emulator, the conditioning circuit, signal processing unit, and corresponding switch groups are included. The ’emulator’ provides some correlative simulation environments for the automatic train operation HILS, the related models included such as accurate braking model, traction transformer model, running line model, velocity fluctuation model, etc. The ’conditioning circuit’ can regulate electrical signals properly for ’Controller’. The ’signal processing unit’ can regulate net signals properly for ’Optimizer’ appropriately.

## 4. Simulation for Automatic Train Operation Tracking Control Scenario

### 4.1. Data and Parameters for Automatic Train Operation Tracking Control Scenario

**Figure 13:**
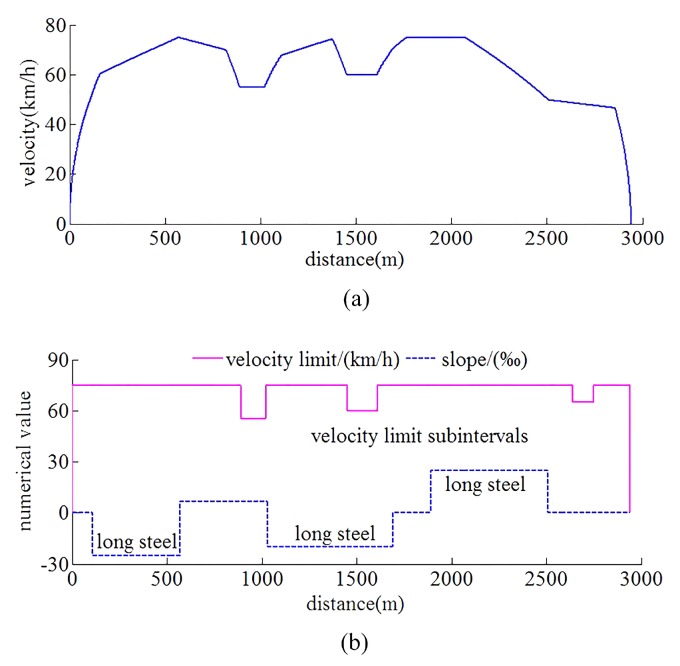
The target velocity trajectory, slopes, and limited velocity curves for automatic train operation tracking control scenario. (**a**) Target velocity trajectory. (**b**) Slopes and limited velocity curves.

The automatic train operation tracking control scenario for rail transit line No.12 in Dalian, China is chosen as the experimental simulation object. Rail transit line No.12 is a significant urban rail transit line with 40.38 kilometers from Hekou station to Lvshun New Port. The running simulation line of scenario about rail transit line No.12 is from Lvshun New Port to Tieshan Town, there are three long steep ramps and three velocity limit subintervals in running interval. The main parameters of the automatic train operation tracking control scenario are shown in Table 3, and the target velocity trajectory, slopes, and limited velocity curves for automatic train operation are shown in Figure 13.

**Figure 14:**
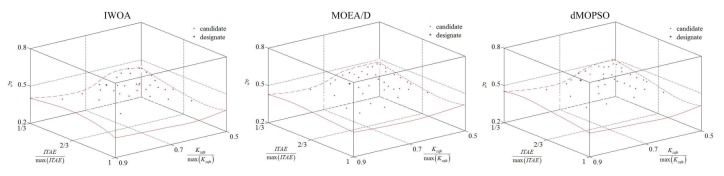
The optimization results for the softness factor adaptive adjusting parameters optimization of each optimization algorithm.

The basic DMC MPC parameters are set as follows; sampling time is 500 μs; model length *N* is 60; control length is 15; predictive length is 15. The addition adjustable parameters for online obtaining of softness factor are set by practical experience as follows; blur width $s_{1}=s_{2}=0.05$ km/h; maximum and minimum value of design expectation $y_{\max}=y_{r}+0.05$ km/h and $y_{\min}\;=\;y_{r}-0.05$ km/h; connected length $S_{1}=S_{2}=0.4$ m. Considering the online real-time calculation efficiency and tracking control effect of the improved DMC MPC proposed in this paper, the following parameters are given based on the relevant scientific literature, field experience, and simulation results of multiple experiments. The Matlab/simulink simulation platform is used for softness factor adaptive adjusting parameters optimization and the experience parameters are as follows; maximum allowed multi-objective performance index, ITAE index, and security index are 0.8, 750, and 6%, respectively; ideal multi-objective performance index, ITAE index, and security index are 0.2, 250, and 3%, respectively. The chosen addition adjustable parameters for online obtaining of softness factor obtained by each optimization algorithms (improved whale optimization algorithm, MOEA/D, dMOPSO) are as follows; fusion weights of $\lambda_{\alpha Ts}$ and $\lambda_{\alpha_{\mu y}}$, $\lambda_{\alpha Ts}=0.582,0.592,0.573$ and $\lambda_{\alpha_{\mu y}}=0.418,0.408,0.427$; maximum value of softness factor $\alpha_{max}$ is 0.939, 0.941, and 0.930; gain coefficient *b* is 0.909, 0.911, and 0.913. The subinterval range obtained by practical experience, reference value of soften factor obtained by each optimization algorithms, synthetic weight of fusion velocity obtained by entropy weight method are shown in Table 4. The specific optimization results (approximate Pareto solution set) for softness factor adaptive adjusting parameters optimization of each optimization algorithms are shown in Table 5 and Figure 14.

In Figure 14, the approximate Pareto solution sets have been obtained by optimization algorithms (IWOA, dMOPSO, and MOEA/D), and one of the approximate Pareto solutions has been chosen for automatic train operation tracking control scenario. As can be seen from Figure 14, compared with other optimization algorithms (dMOPSO and MOEA/D), the wider dominated portion for approximate Pareto solution sets obtained by using IWOA. This indicates that the IWOA proposed in this paper has better optimization effectiveness. As can be seen from Table 5, compared with other optimization algorithms (dMOPSO and MOEA/D), IWOA has been improved to a considerable extent not only in the computation speed but also in the optimization effect for approximate Pareto solution set reflected by the hypervolume indicator ratio $\frac{{I^{*}}_{H}\left(A\left(op\right)\right)}{V\left(\Omega_{S}\right)}$. $V\left(\Omega_{S}\right)$ is the volume of selected objective space $\Omega_{S}$ by experience, and it is 0.16 ($\frac{3}{5}\times \frac{2}{3}\times \frac{2}{5}$) in this paper.

### 4.2. Matlab/simulink Simulation Results for Automatic Train Operation Tracking Control Scenario

**Figure 15:**
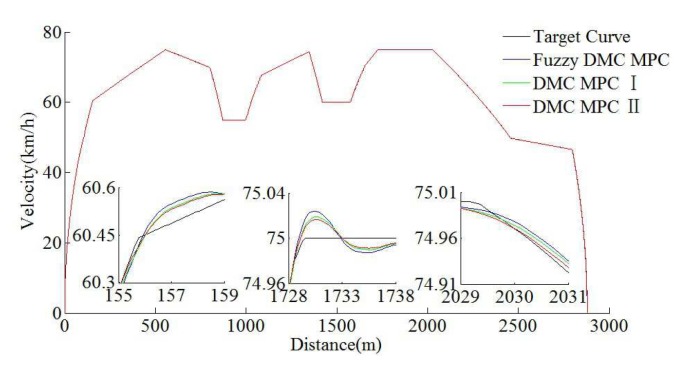
The Matlab/simulink velocity trajectory curves of different DMC MPC algorithms for automatic train operation tracking control scenario.

**Figure 16:**
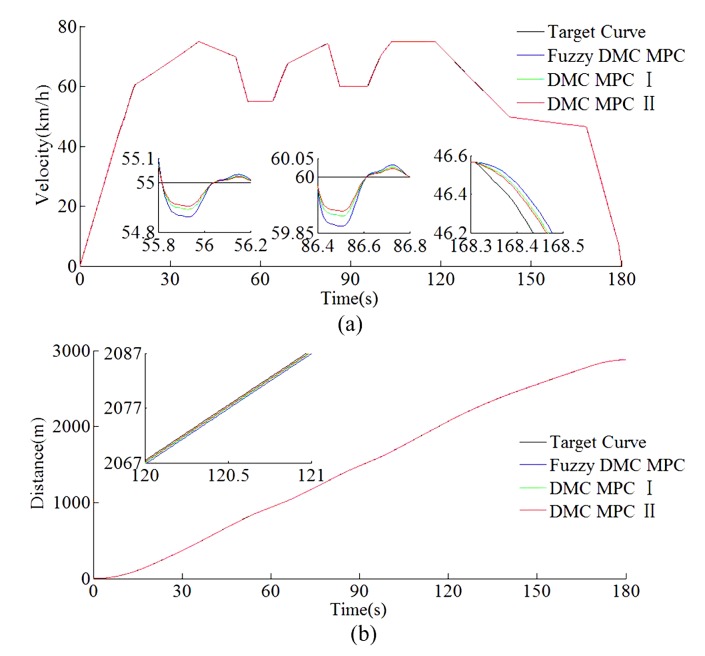
The Matlab/simulink time traceability curves of different DMC MPC algorithms for automatic train operation tracking control scenario. (**a**) Time–velocity curves. (**b**) Time–distance curves.

**Figure 17:**
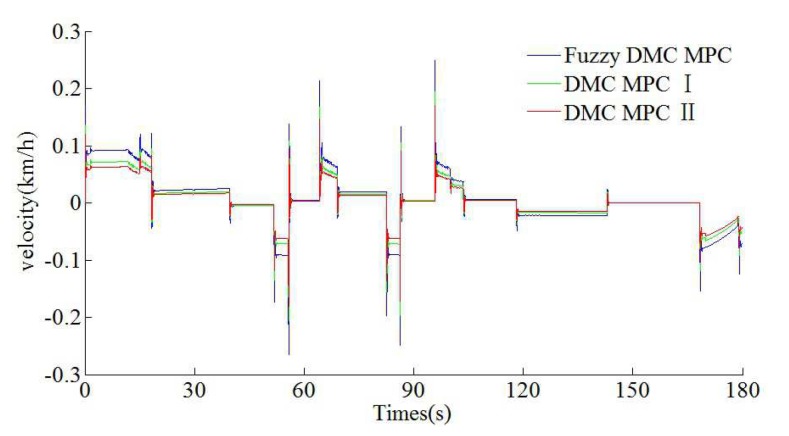
The Matlab/simulink time velocity error curves of different DMC MPC algorithms for automatic train operation tracking control scenario.

**Figure 18:**
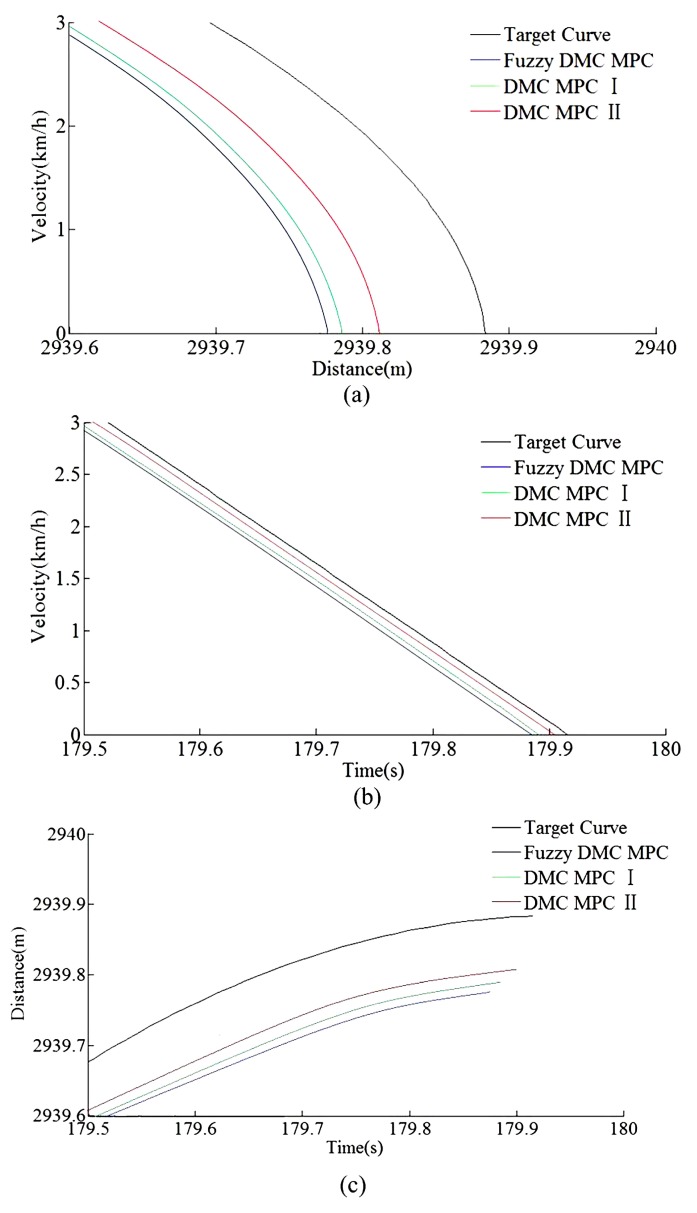
The Matlab/simulink parking error curves of different DMC MPC algorithms for automatic train operation tracking control scenario. (**a**) Distance–velocity curves. (**b**) Time–velocity curves. (**c**) Time–distance curves.

The sampling process is not necessary in the Matlab/simulink simulation platform. According to the automatic train operation tracking control scenario of rail transit line No.12 in Dalian, China, the Matlab/simulink simulation results are obtained by using the fuzzy DMC MPC based on online obtaining of softness factor α, and the softness factor adaptive adjusting parameters optimization using IWOA proposed in this paper (denoted as DMC MPC II); the fuzzy DMC MPC is based on online obtaining of softness factor α, which uses softness factor adaptive adjusting parameters optimization using MOEA/D (denoted as DMC MPC I) and traditional fuzzy DMC MPC. The specific configuration of the Matlab/simulink platform used in this paper is described as follows; the Matlab/simulink revision is 2016b, MathWorks; the type of computer processor is CPU Core i9-7920X @ 2.9GHZ. The specific Matlab/simulink results are shown in Figure 15, Figure 16, Figure 17 and Figure 18 and Table 6 and Table 7.

As can be seen from Table 6 and Table 7, the tracking control results obtained by the DMC MPC II are superior to that of DMC MPC I and traditional fuzzy DMC MPC, and four indexes of multi-objective performance index (energy saving, punctuality, parking precision, and comfort), ITAE index, and security index for automatic train operation have been improved considerably. As can be seen from the Figure 15 and Figure 16, the DMC MPC II can make the tracking control curves closer to target curves, so as to obtain the ideal tracking control results as smooth as possible. As can be seen from the six enlarged areas of the velocity trajectory curves in Figure 15 and Figure 16, the velocity fluctuation degree is weaker and the velocity trajectory is closer to the target by using DMC MPC II. As can be seen from the one enlarged areas of time distance curves of Figure 16, compared with DMC MPC I and traditional fuzzy DMC MPC, the time traceability of DMC MPC II is more powerful, so as to obtained the time distance curve closer to target. As can be seen from Figure 17, the smaller velocity error effect can be obtained by using DMC MPC II. As can be seen from Figure 18, the more ideal parking results can be obtained by using DMC MPC II; both the distance and time errors of parking are reduced to certain extent, so as to improve the punctuality and fixed position effect.

Compared with traditional fuzzy DMC MPC and DMC MPC I, DMC MPC II has several obvious superiorities in the matlab/simulation environment. However, as there is no hardware equipment in the actual automatic train operation tracking control scenario in matlab/simulation environment, the effectiveness of DMC MPC proposed in this paper must be further tested and verified.

### 4.3. HILS Results for Automatic Train Operation Tracking Control Scenario

**Figure 19:**
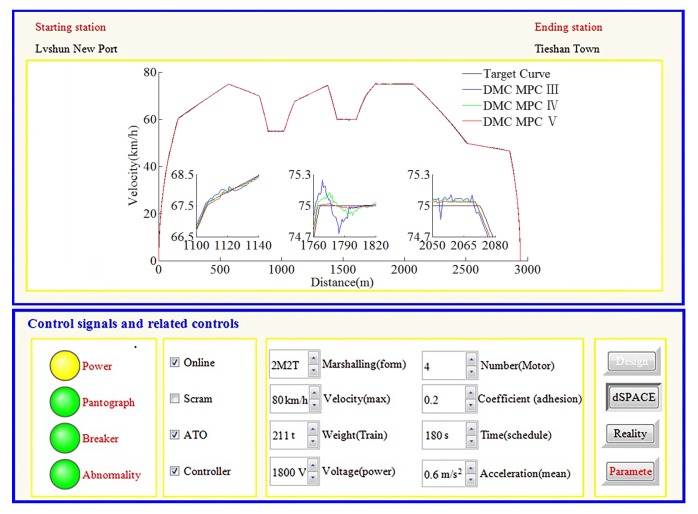
The HILS velocity trajectory curves of different DMC MPC algorithms for automatic train operation tracking control scenario.

**Figure 20:**
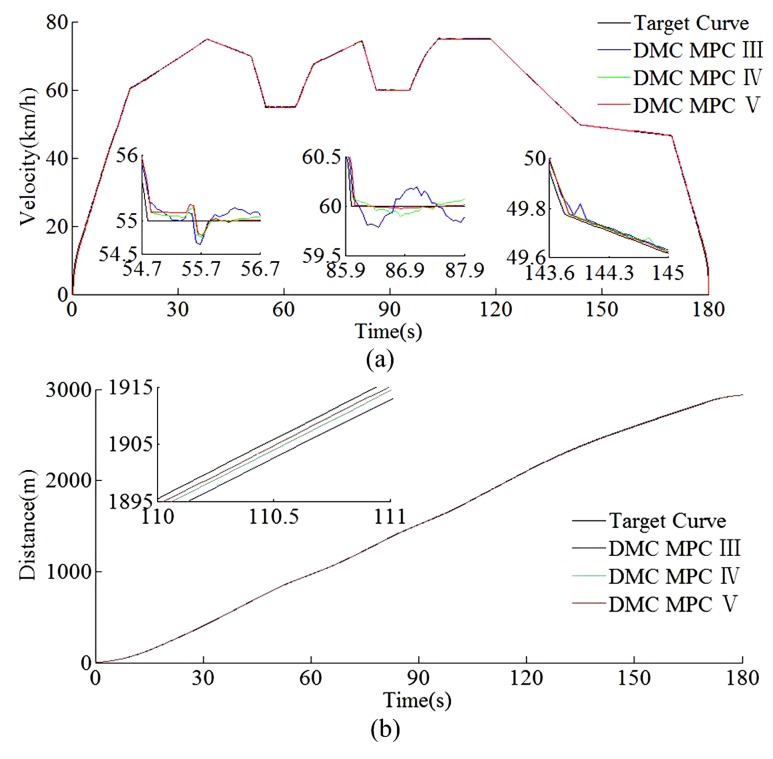
The HILS time traceability curves of different DMC MPC algorithms for automatic train operation tracking control scenario. (**a**) Time–velocity curves. (**b**) Time–distance curves.

**Figure 21:**
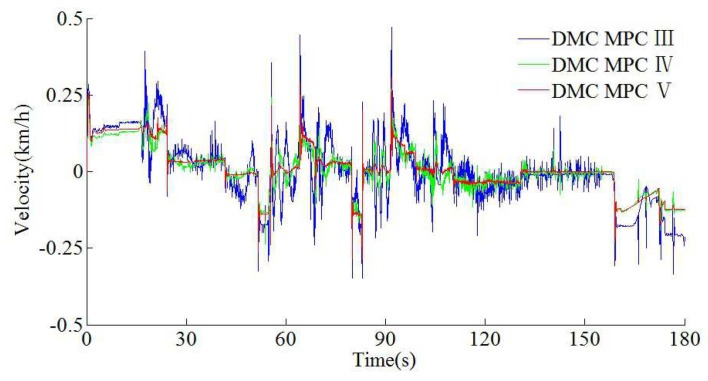
The HILS time–velocity error curves of different DMC MPC algorithms for automatic train operation tracking control scenario.

**Figure 22:**
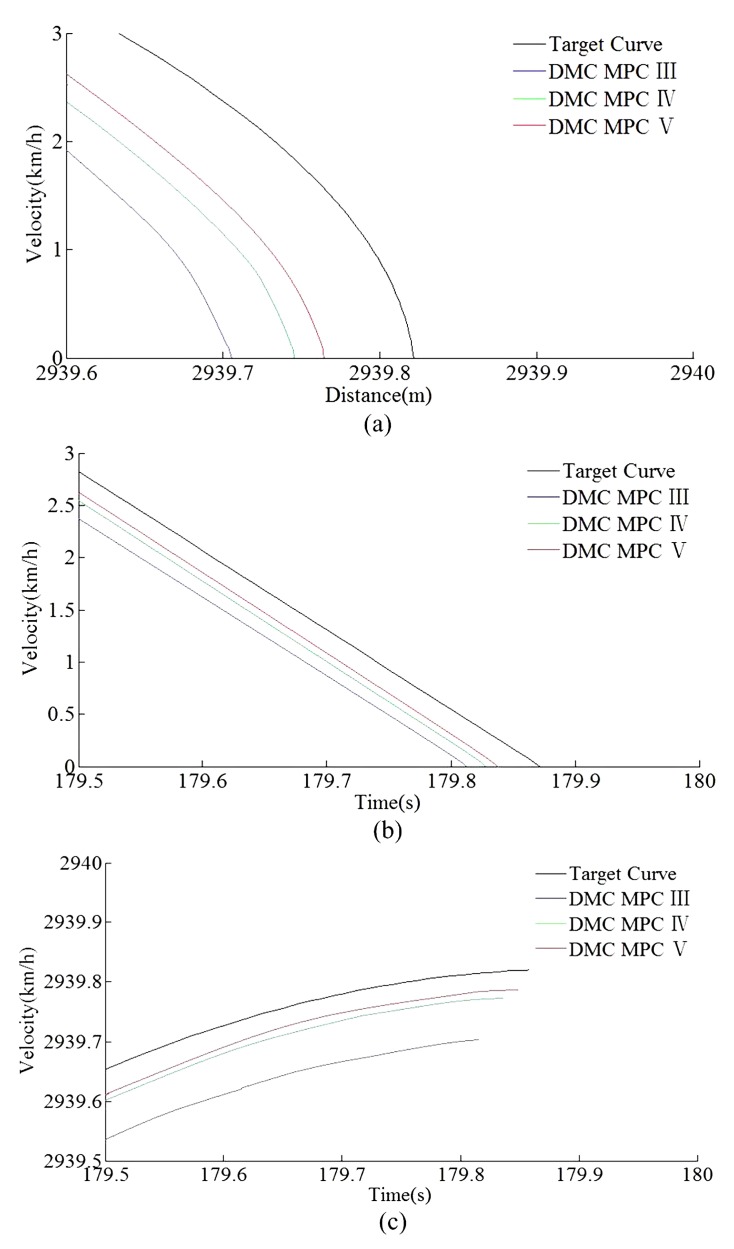
The HILS parking error curves of different DMC MPC algorithms for automatic train operation tracking control scenario. (**a**) Distance–velocity curves. (**b**) Time–velocity curves. (**c**) Time–distance curves.

In this way, sampling accuracy must be taken into account. To further verify the effectiveness of the algorithm, according to the automatic train operation tracking control scenario of rail transit line No.12 in Dalian, China, the HILS results are obtained by using the fuzzy DMC MPC based on online obtaining of softness factor α and fusion velocity, which uses the softness factor adaptive adjusting parameters optimization using IWOA proposed in this paper (denoted as DMC MPC V), the fuzzy DMC MPC based on online obtaining of softness factor α and fusion velocity, which softness factor adaptive adjusting parameters optimization using dMOPSO (denoted as DMC MPC IV) and the fuzzy DMC MPC based on online obtaining of fusion velocity (denoted as DMC MPC III). The specific configuration of the automatic train operation HILS platform used in this paper is described as follows; the Matlab/simulink revision is “2016b, MathWorks”; the type of computer processor is “CPU Core i9-7920X @ 2.9GHZ”; the core chip of “Tracking controller” and “Motor optimizer” is “TMS320F28335”; the simulation software of “dSPACE emulator” is dSPACE control desk (revision is control desk 6.1); the communication protocol of the HILS platform is MVB (multifunction vehicle bus); the fuzzy PID (proportion integration differentiation) algorithm is adopted as motor control algorithm; vehicle velocity proportion is (0.83 × 400 rad/min)/(80 km/h). The specific HILS results are shown in Figure 19, Figure 20, Figure 21 and Figure 22 and Table 8 and Table 9.

According to the HILS results of different algorithms from Table 8 and Table 9, compared with DMC MPC III and DMC MPC IV, DMC MPC V has an obvious performance improvement effectiveness, the multi-objective performance index (energy saving, punctuality, parking precision, and comfort) of the tracking control trajectory has been improved considerably; meanwhile, the ITAE index and security index have also been reduced considerably. In Figure 19, during the automatic train operation tracking control experiment simulation, all the pilot lights and buttons are in normal. As can be seen from Figure 19 and Figure 20, the DMC MPC V can bring the tracking control curves closer to the target curves, so as to obtain the ideal tracking control results as smooth as possible. As can be seen from the six enlarged areas of velocity trajectory curves of Figure 19 and Figure 20, the velocity trajectory curves obtained by DMC MPC V were smoother; compared with the traditional improved tracking control algorithm (DMC MPC), DMC MPC V enables the train to be in the optimal working state as much as possible, so as to reduce the velocity fluctuation degree and obtain more ideal tracking control results. As can be seen from the one enlarged areas of the time–distance curves in Figure 20, compared with DMC MPC III and DMC MPC IV, the time traceability of DMC MPC V is more powerful, so as to obtain a time–distance curve closer to target. As can be seen from Figure 21, the velocity error obtained by using DMC MPC V is smaller. As can be seen from Figure 22, the more ideal parking point (parking time and position) can be obtained by using DMC MPC V, its parking point is closer to prospective parking point (180 s and 2940 m).

The above HILS results show that DMC MPC V is a tracking control algorithm with good practical tracking control effect for automatic train operation tracking control scenario.

## 5. Conclusions

Tracking control optimization for automatic train operation is a sophisticated optimization problem, and the model predictive controller is widely used to solve this problem due to its advantages of strong robustness and good performance in tracking speed and tracking precision. Aiming at obtaining a more ideal tracking control performance for automatic train operation, an improved model predictive control algorithm and corresponding controller based on online obtaining of softness factor and fusion velocity for automatic train operation are proposed and developed, and an improved whale optimization algorithm based on Tchebycheff decomposition method was proposed for softness factor adaptive adjusting parameters optimization. This clearly shows that model predictive controller has been improved to a considerable extent in tracking control optimization for automatic train operation not only in the pure software scenarios but also in the hardware-in-the-loop simulation scenarios (the ITAE index obtained by DMC MPC II is 16.3% and 31.2% lower than that of DMC MPC I and Fuzzy DMC MPC, and that obtained by the DMC MPC V is 7.3% and 16.1% lower than that of DMC MPC IV and DMC MPC III). The specific advantages are described below.

(I) Aiming at improving the efficiency of the whale optimization algorithm based on the Tchebycheff decomposition method, the strategy of cosine decline combined with chaotic random method for convergence factor nonlinear decline is proposed, so as to obtain more satisfactory softness factor adaptive adjusting parameters for tracking control.
(II) Not only is an improved online adaptive adjusting method for softness factor based on fuzzy satisfaction of system output value and velocity distance trajectory characteristic adopted, but also a fusion velocity model and a corrected model of real-time sampling for automatic train operation tracking control are adopted. Thus, compared with traditional improved model predictive controller, the improved model predictive controller developed in this paper based on online obtaining of softness factor and fusion velocity could enable the train in the optimal working state as much as possible, so as to obtain a more ideal tracking control result with more satisfactory performance indexes, including energy saving, punctuality, parking precision and comfort, ITAE, and security index.
(III) For any tracking control system, the accomplishing capacity of computational tasks real-time is significant important. The only purpose of the improved strategies is the online obtaining of optimal softness factor and fusion velocity, so as to obtain the more reasonable real-time control quantity u(k), and enable the automatic train operation tracking control system robustness and rapidity as much as possible. Thus, the quantity of additional computational tasks is not very large. In addition, some complex computational tasks for adjustable parameters optimization for softness factor adaptive adjusting and setting synthetic weight of the velocity sampled according to entropy weight method are achieved offline. Then, the advanced urban rail vehicle velocity monitoring device and the additional function chip of online obtaining of softness factor and fusion velocity are applied to improved the accomplishing capacity of computational tasks real-time is significant important. Finally, some complex functions such as logarithm or exponential function are avoided in online computation. Thus, it has advantage of simple and easily conducted.

According to the Matlab/simulink results and ATO HILS results (compare with the other DMC MPC algorithms for comparison), the improved DMC MPC based on online obtaining of softness factor and fusion velocity proposed in this paper has better tracking control performance, so it can obtain more ideal tracking control results. In actuality, it can also be designed by other ways for online obtaining of softness factor and fusion velocity. It is important to note that, as the computational tasks such as various matrix computation of basic DMC MPC are onerous, the designed scheme should be simple and appropriate caused by the limited computation margin in real-time.

## Figures and Tables

**Table 1:** The hypervolume indicator ratio $\frac{{I^{*}}_{H}\left(A\left(op\right)\right)}{{I^{*}}_{H}\left(A\left(tp\right)\right)}$ for test functions of each optimization algorithm.

Optimization Algorithm	ZDT1	ZDT1	DTLZ2
IWOA	99.81%	99.73%	99.04%
MOEA/D	99.72%	99.60%	98.73%
dMOPSO	99.54%	99.37%	98.57%

**Table 2:** The computation time for test functions of each optimization algorithm.

Optimization Algorithm	ZDT1	ZDT1	DTLZ2
IWOA	1279*s*	1541*s*	1895*s*
MOEA/D	1384*s*	1733*s*	2079*s*
dMOPSO	1423*s*	1694*s*	2104*s*

**Table 3:** The main parameters of the automatic train operation tracking control scenario.

Parameter Name	Parameter Characteristics
Maximum limited velocity (km/h)	75
Running interval distance (m)	2940
Prospective running time (s)	180
Maximum allowed parking error (m)	0.4
Maximum allowed punctual time error (s)	0.5

**Table 4:** The optimization results for subinterval range, reference value of soften factor $\alpha_{Tr}$, and synthetic weight of fusion velocity $\lambda_{is}$.

Subinterval Index	Subinterval Range *s* (m)	$\alpha_{\mathrm{Tr}}$ Obtained by (IWOA, MOEA/D, dMOPSO)	Synthetic Weight $\lambda_{\mathrm{is}}$
1	0–140	0.892, 0.896, 0.897	0.76, 0.15, 0.09
2	140–210	0.924, 0.926, 0.919	0.20, 0.71, 0.09
3	210–753	0.885, 0.883, 0.882	0.83, 0.11, 0.16
4	753–830	0.924, 0.922, 0.926	0.20, 0.61, 0.19
⋯	⋯	⋯	⋯
14	1430–1490	0.930, 0.928, 0.927	0.33, 0.51, 0.16
⋯	⋯	⋯	⋯
24	2910–2940	0.914, 0.911, 0.912	0.09, 0.08, 0.83

**Table 5:** The hypervolume indicator ratio $\frac{{I^{*}}_{H}\left(A\left(op\right)\right)}{V\left(\Omega_{S}\right)}$ and computation time for softness factor adaptive adjusting parameters optimization of each optimization algorithms.

Optimization Algorithm	Computation Time	Hypervolume Indicator Ratio
IWOA	3726*s*	60.94%
MOEA/D	4582*s*	63.37%
dMOPSO	5047*s*	71.84%

**Table 6:** The Matlab/simulink tracking control results of energy saving, punctuality, parking precision, and comfort for automatic train operation.

Algorithm	Energy Consumption	Actual Time	Parking Position	Comfort Level
Target curve	98615 KJ	179.914 s	2939.884	5.517 m/s${}^{2}$/km
Fuzzy DMC MPC	112094 KJ	179.871 s	2939.774	30.725 m/s${}^{2}$/km
DMC MPC I	110844 KJ	179.892 s	2939.781	28.754 m/s${}^{2}$/km
DMC MPC II	108759 KJ	179.032 s	2939.812	24.339 m/s${}^{2}$/km

**Table 7:** The Matlab/simulink tracking control results of multi-objective performance index, ITAE index, and security index for automatic train operation.

Algorithm	Multi-Objective Performance Index	ITAE Index	Security Index
Fuzzy DMC MPC	0.507	552.69	5.12%
DMC MPC I	0.467	454.87	4.89%
DMC MPC II	0.370	380.54	4.63%

**Table 8:** The HILS tracking control results of energy saving, punctuality, parking precision, and comfort for automatic train operation.

Algorithm	Energy Consumption	Actual Time	Parking Position	Comfort Level
Target curve	98703 KJ	179.874 s	2939.823	5.429 m/s${}^{2}$/km
DMC MPC III	119,192 KJ	179.809 s	2939.704	35.403 m/s${}^{2}$/km
DMC MPC IV	115,048 KJ	179.824 s	2939.754	32.935 m/s${}^{2}$/km
DMC MPC V	113,784 KJ	179.835 s	2939.778	31.354 m/s${}^{2}$/km

**Table 9:** The HILS tracking control results of multi-objective performance index, ITAE index, and security index for automatic train operation.

Algorithm	Multi-Objective Performance Index	ITAE Index	Security Index
DMC MPC III	0.712	821.57	5.68%
DMC MPC IV	0.585	744.23	5.14%
DMC MPC V	0.530	690.43	5.09%

## Abbreviations

The following abbreviations are used in this manuscript.

ATO	automatic train operation
HILS	hardware-in-the-loop simulation
DMC MPC	dynamic matrix control model predictive control
DMC MPC I	fuzzy DMC MPC based on online obtaining of softness factor which softness factor adaptive adjusting parameters optimization using MOEA/D
DMC MPC II	fuzzy DMC MPC based on online obtaining of softness factor which softness factor adaptive adjusting parameters optimization using IWOA
DMC MPC III	fuzzy DMC MPC based on online obtaining of fusion velocity
DMC MPC IV	fuzzy DMC MPC based on online obtaining of softness factor and fusion velocity which softness factor adaptive adjusting parameters optimization using dMOPSO
DMC MPC V	fuzzy DMC MPC based on online obtaining of softness factor and fusion velocity which softness factor adaptive adjusting parameters optimization using IWOA
